# Structural Mechanism of Receptor-Triggered MyD88 Oligomeric Assembly in Innate Immune Signaling

**DOI:** 10.1038/s41467-026-71836-8

**Published:** 2026-04-17

**Authors:** Kazuki Kasai, Kayo Imamura, Masatoshi Uno, Shiho Nukui, Naotaka Sekiyama, Tomoko Miyata, Fumiaki Makino, Ryusei Yamada, Yoshiki Takahashi, Noriyuki Kodera, Keiichi Namba, Hidenori Ohnishi, Akihiro Narita, Hiroki Konno, Hidehito Tochio

**Affiliations:** 1 Department of Biophysics, Graduate School of Science, Kyoto University, Kitashirakawa Oiwake-cho, Sakyo-ku, Kyoto, Japan; 2 Graduate School of Frontier Biosciences, The University of Osaka, 1-3 Yamadaoka, Suita, Osaka, Japan; 3 JEOL YOKOGUSHI Research Alliance Laboratories, The University of Osaka, 1-3 Yamadaoka, Suita, Osaka, Japan; 4 JEOL Ltd., Akishima, 3-1-2 Musashino, Akishima, Tokyo, Japan; 5 Graduate School of Natural Science and Technology, Kanazawa University, Kakuma-cho, Kanazawa, Ishikawa, Japan; 6 WPI Nano Life Science Institute (WPI-NanoLSI), Kanazawa University, Kakuma-cho, Kanazawa, Ishikawa, Japan; 7 Department of Pediatrics, Graduate School of Medicine, Gifu University, 1-1 Yanagido, Gifu, Japan; 8 Laboratory of Intractable and Rare Diseases, Graduate school of medicine, Gifu University, 1-1 Yanagido, Gifu, Japan; 9 Center for One Medicine Innovative Translational Research (COMIT), Gifu University, 1-1 Yanagido, Gifu, Japan; 10 Department of Biological Science, Graduate School of Sciences, Nagoya University, Furo-cho, Chikusa-ku, Nagoya, Japan

**Keywords:** Cryoelectron microscopy, Supramolecular assembly, Atomic force microscopy, Toll-like receptors, Interleukins

## Abstract

MyD88 plays a pivotal role in Toll-like receptor (TLR) and interleukin-1 family signaling through its oligomerization upon receptor activation, leading to downstream protein recruitment. The Toll/interleukin-1 receptor domain of MyD88 (TIR_MyD88_) is responsible for this receptor-mediated oligomerization, but the detailed mechanism involved remains elusive. Here we investigate the structure of TIR_MyD88_ oligomers and their interactions with TLRs. Cryoelectron microscopy reveals that tandemly arrayed TIR_MyD88_ subunits form an antiparallel double-stranded filament that can further form rings and cylindrical filaments. Moreover, the self-assembly of TIR_MyD88_ in vitro is markedly accelerated by dimeric rather than monomeric receptor TIRs, possibly reflecting the signal initiation step in vivo. High-speed atomic force microscopy further captures the dynamic processes of oligomerization of TIR_MyD88_, in addition to its direct interaction with the receptor TIRs. These results reveal a regulatory mechanism of TIR_MyD88_ oligomerization underlying the signal initiation step.

## Abstract (web summary)

MyD88 is an immune adaptor protein that self-assembles via its TIR domain to initiate TLR and inflammatory signaling. Here, authors reveal the cryo-EM structure of MyD88 TIR filaments and show how dimerized receptors directly nucleate this assembly.

## Introduction

Myeloid differentiation primary response 88 (MyD88) is the main cytosolic adaptor protein involved in innate immune and inflammatory signaling^1,2^. It interacts with interleukin-1 receptor (IL-1R) family members and most members of the Toll-like receptor (TLR) family. Upon ligand binding, these receptors interact with MyD88, which subsequently recruits and activates members of the IL-1R-associated kinase (IRAK) family, such as IRAK4 and IRAK1.

MyD88 consists of two protein‒protein interaction domains: the death domain (DD_MyD88_) at the N-terminus and the Toll/IL-1R domain (TIR_MyD88_) at the C-terminus. These domains are connected by a linker of 46 amino acid residues called the intermediate domain (ID)^3,4^. Studies have shown that MyD88 has intramolecular interactions between DD_MyD88_ and TIR_MyD88_, which may play a regulatory role in signal transduction^5,6^. On the other hand, in general, both the DD and TIR domains participate in homotypic interactions, leading to the formation of oligomeric signaling complexes^2,7^. During IL-1R and TLR signaling, DD_MyD88_ forms helical homo-oligomers^8^ that serve as scaffolds for the successive recruitment of IRAK4 and IRAK1/2. The details of the interaction governing this recruitment were clarified by the determination of the tripartite complex structure of DD_MyD88_/DD_IRAK4_/DD_IRAK2_^9^, in which a three-layer DD oligomer is formed by the respective DDs at a 6:4:4 ratio. It is speculated that the formation of this DD oligomer brings the kinase domains of the IRAKs into close proximity, facilitating transphosphorylation, which triggers their activation and the subsequent phosphorylation of downstream proteins, such as TRAF6^2^.

While DD_MyD88_ is responsible for recruiting and activating IRAKs, TIR_MyD88_ plays a crucial role in interacting with IL-1Rs/TLRs through homotypic TIR-TIR interactions. Signaling by these receptors is initiated by ligand-induced dimerization of their extracellular domains, which leads to subsequent dimerization of their cytosolic TIR domains. The dimerized receptor TIRs then engage with TIR_MyD88_, triggering the formation of MyD88 clusters at the receptor site^10^. In some but not all TLR signaling modes, another adaptor protein, MyD88 adaptor-like protein (Mal), plays an essential role by mediating interactions between TLRs and MyD88^11,12^. A recent live-cell imaging study revealed that in IL-1 signaling, the formation of MyD88 clusters of a certain size is necessary for the recruitment of IRAK4 and the initiation of appropriate signaling. Thus, the authors proposed that such clustering functions as a “physical threshold” for signaling^13^.

Although the monomeric structure of TIR_MyD88_ was reported years ago^14,15^, the manner in which TIR_MyD88_ oligomerizes has remained elusive. Recently, Ve et al. reported that TIR_MyD88_ forms a fibrous structure in the presence of the TIR domain of Mal (TIR_Mal_)^16^. Clabbers et al. subsequently grew crystals of TIR_MyD88_ by seeding with filaments of TIR_Mal_^17^, demonstrating that in the crystals, the TIR_MyD88_ subunits were arrayed in a manner identical to that observed for the subunits in the seeded TIR_Mal_ filaments. Therefore, the authors propose a molecular templating mechanism, in which TIR_Mal_ assemblies act as templates for the assembly of TIR_MyD88_. Although such templating may be a role for TIR_Mal_, it is important to note that Mal is not required for many of the MyD88-dependent signals. For instance, while Mal is essential for a subset of TLRs (such as TLR2 and TLR4)^2,12^, it is not required for signaling through other TLRs, such as TLR5 and TLR7. Even TLR2 can initiate MyD88-dependent signaling without Mal^18^. Furthermore, the IL-1 family (IL-1α, IL-1β, IL-18 and IL-33) does not require Mal for MyD88-dependent signaling^19,20^. In such cases, Mal-templated oligomerization cannot occur, and thus the receptors directly trigger the self-assembly of TIR_MyD88_. Therefore, for a comprehensive understanding of MyD88-dependent signaling, it is crucial to elucidate the self-assembly mode of TIR_MyD88_ in the absence of Mal. Furthermore, the detailed molecular process by which the receptors trigger TIR_MyD88_ assembly remains elusive. In addition to these mechanistic interests, the self-assembly mode of TIR_MyD88_ is of clinical importance. In aggressive B-cell lymphoma subtypes, somatic gain-of-function mutations in TIR_MyD88_, such as L252P (historically L265P), are frequently identified. These mutations lead to an unusually pronounced self-assembly of MyD88 independent of receptors as well as Mal, resulting in aberrant activation of the NF-κB and MAP kinase pathways. This unregulated activation is a primary cause of increased tumor malignancy^21–23^. Additionally, these mutations are implicated in autoinflammatory diseases and severe arthritis^24,25^. Therefore, the molecular details of the TIR_MyD88_ self-assembly process may also provide valuable insights into the pathological mechanisms underlying these diseases and facilitate the development of new therapeutics.

Here, we show that even in the absence of Mal, TIR_MyD88_ self-assembles into filaments that can further form ring structures and cylindrical fibers. Notably, the addition of a small amount of dimeric receptor TIRs markedly promotes the self-assembly of TIR_MyD88_, consistent with the physiological role of receptor dimerization in vivo. Through cryoelectron microscopy (cryo-EM), we reveal the self-assembled structure of TIR_MyD88_, which differs significantly from previously reported Mal-induced TIR_MyD88_ oligomers, yet shows partial similarity, providing vital functional insights. We further visualize the dynamics of the TIR_MyD88_ oligomerization and capture its direct binding to receptor TIRs via high-speed atomic force microscopy (HS-AFM), demonstrating that a specific loop reorganization acts as a kinetic barrier for oligomerization of TIR_MyD88_. Combining these results with those of structure-guided cell-based assays, we propose a unique regulatory mechanism for the receptor-triggered assembly of MyD88, providing molecular insights into the formation of Myddosome signaling complex^9^ and related pathogenetic mechanisms. This study elucidates the mechanistic basis of a previously uncharacterized Mal-independent route to MyD88 activation, wherein receptor dimerization directly nucleates TIR_MyD88_ filaments that are structurally distinct from Mal-induced assemblies.

## Results

### Self-assembled oligomers of TIR_MyD88_

**Fig. 1:**
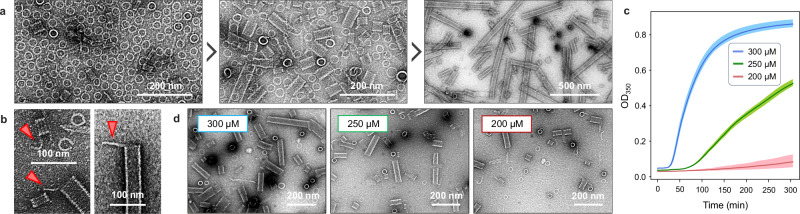
Time course of TIR_MyD88_ self-assembly. **a** Negative stain TEM images of TIR_MyD88_ (200 μM) recorded after different incubation times (2 h, 1 day, and 3 days) at 30 °C. **b** The free filaments projected from the cylindrical fibers are indicated by red triangles. **c** A turbidity assay was performed in triplicate at 37 °C with different concentrations of TIR_MyD88_. The optical density at 350 nm (OD_350_) was measured every 5 min. Data are presented as the mean turbidity of triplicate experiments. Shaded bands indicate the range between minimum and maximum values. **d** TEM images of fiber samples collected after the turbidity assay described in (**c**). Representative images from more than five independent experiments with consistent results are shown. Source data are provided as a Source Data file.

We expressed and purified a recombinant TIR domain of MyD88 (TIR_MyD88_; residues 153–296) and found that it spontaneously assembled into filaments when incubated in purification buffer (10–300 μM). The filaments also formed rings with a characteristic diameter of 22–38 nm (Fig. 1a, left) and, occasionally, larger rings with a diameter of 60–80 nm. Moreover, as the incubation time increased, short cylindrical fibers were formed (Fig. 1a, middle), resulting in a predominance of long, straight cylindrical fibers (Fig. 1a, right). Although the global morphology varied with incubation time (rings or cylinders), all morphologies appeared to form from the common filaments observed during early incubation. This conclusion was based on the observation that such filaments projected from disordered/degraded cylindrical structures (Fig. 1b). Thus, we considered the filaments to be fundamental for the formation of the rings and cylindrical fibers. To characterize the time course of TIR_MyD88_ self-assembly, turbidity assays were conducted, and the results showed a sharp increase in the assembly rate for TIR_MyD88_ concentrations greater than around 200 μM (Fig. 1c, d). Whereas we found that TIR_MyD88_ formed fibers at concentrations of 10 μM or above, it was previously reported that it had no tendency to form fibrils by itself^16^. One possible explanation for this inconsistency is the presence of additional amino acid residues, including a purification tag (His-tag) at the C-terminus of TIR_MyD88_, as reported in the previous study. These additional residues may have perturbed the intricate intermolecular interactions, preventing TIR_MyD88_ self-assembly.

### HS-AFM reveals that TIR_MyD88_ can form ring structures

**Fig. 2:**
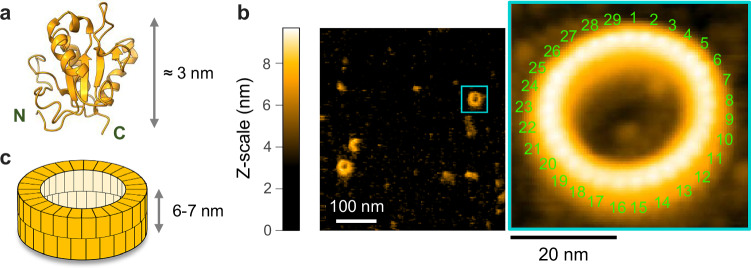
AFM images of TIR_MyD88_ rings. **a** Ribbon model of monomeric TIR_MyD88_ (PDB ID: 2Z5V). **b** Left: representative overview of the rings as visualized by AFM. Scan area: 500 × 500 nm^2^ with 150 × 150 pixels. Scan speed: 600 ms per image. Right: magnified view of the ring enclosed in the cyan square in (**b**). Scan area: 60 × 60 nm^2^ with 120 × 120 pixels. Scan speed: 600 ms per image. The image was processed with low-pass filtering and 20-frame averaging. **c** Schematic of the ring shown in (**b**). Representative images from more than five independent experiments with consistent results are shown.

To determine whether TIR_MyD88_ forms filaments, we employed HS-AFM^26,27^, which enables single-molecule visualization of biomacromolecules in physiologically relevant aqueous solutions. Although only a sparse distribution of monomeric TIR_MyD88_ was observed in diluted solution (10 nM)^5^, incubation with higher concentrations (10–200 μM) resulted in the formation of ring structures, with sizes and shapes similar to those observed by TEM, within 10 min (Fig. 2b, c). The height and diameter of the rings were 6–7 nm and 24–36 nm (average, 29 nm), respectively (Fig. 2 and Supplementary Fig. 1). Examination of the nodules on the surface of one typical ring led to the identification of 29 subunits (each ~3 nm in diameter) on the surface (Fig. 2a, b). Because the height of the ring corresponded to that of two monomers of TIR_MyD88_, the ring was anticipated to be double layered, indicating that it contained a total of 58 subunits (Fig. 2c). The double-layered structure was evident in cross sections of a partially formed ring. The rings contained two distinct regions with different heights, i.e., 6.5 nm and 3.2 nm (Supplementary Fig. 1), corresponding to double- and single-layered regions, respectively.

Notably, we did not observe cylindrical fibers by AFM, as we did by TEM (Fig. 1). In AFM, two types of forces are applied to a sample; thus, only molecular assemblies that are resistant to these forces are observed. One such force is between the sample and the substrate, and the other is between the sample and the AFM tip. The former force is difficult to estimate, but it can sometimes be strong enough to break biomolecular complexes^28,29^. In contrast, the latter force can be estimated to be approximately 20–30 pN based on the properties and amplitude of the cantilever during observation^30^, which is relatively small and unlikely to disrupt the assemblies. Therefore, it is most likely that the former force was responsible for preventing the formation of cylindrical fibers.

### Determination of the structure of the cylindrical fibers of TIR_MyD88_ by cryo-EM

The ability of TIR_MyD88_ to form filaments and higher-order assemblies under physiological conditions suggests that the observed self-assembly mode represents a functional state of TIR_MyD88_ in cells. Furthermore, gain-of-function mutations identified in TIR_MyD88_ have been reported to be relevant to refractory B-cell lymphomas, such as activated B-cell-like diffuse large B-cell lymphoma (ABC-DLBCL)^21^, as well as a subset of Schnitzler’s syndrome cases^24^. These mutations, particularly L252P (historically L265P), have been shown to result in aberrant self-assembly of TIR_MyD88_ and constitutively activate downstream signals that promote tumor survival^21,22^. Given the anticipated functional and clinical significance of the self-assembled structure of TIR_MyD88_, we sought to determine the structure of the cylindrical fibers using cryo-EM.

**Fig. 3:**
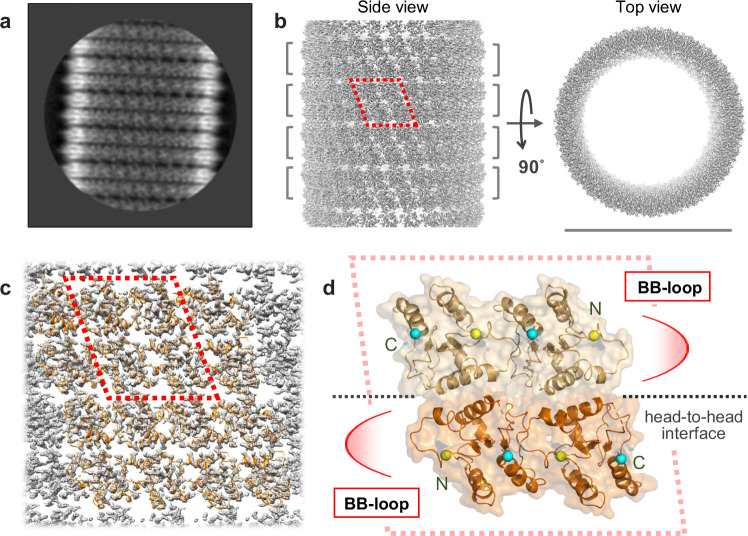
Cryo-EM structure of the cylindrical fibers of TIR_MyD88_. **a** Representative image of a cylindrical fiber of self-assembled TIR_MyD88_ after 2D class averaging. **b** Cryo-EM density map of the cylindrical fiber in two orientations. The gray brackets indicate the positions of the double-layered rings. The gray bar under the top view represents a length of 28 nm. **c** Molecular models fitted to the EM map. **d** Depiction of the four subunits enclosed in the red dotted outlines in (**b**) and (**c**) in ribbon and surface representations. The red arcs and black dotted lines indicate the intrastrand and head-to-head stacking interfaces, respectively. The N- and C-termini are depicted as yellow and cyan spheres, respectively.

The obtained micrographs showed cylindrical fibers with diverse widths. Two types of frequently observed cylinders were selected for analysis: thinner cylinders (~28 nm diameter) and thicker cylinders (~36 nm diameter) (Supplementary Figs. 2a, 7a). We first analyzed the thinner cylinders. After 2D class averaging, the images clearly show double-layered rings stacked coaxially (Fig. 3a). This structure is reminiscent of the double-layered rings observed by AFM. The height of each double ring in the cylinder was 6.6 nm, which was consistent with that of the rings observed by AFM (Fig. 2 and Supplementary Fig. 1). Next, 3D helical reconstruction was performed on the 26,680 selected images, resulting in a 3D EM map with a resolution of 3.3 Å (FSC 0.143), and an atomistic model was constructed (Fig. 3b, c, Supplementary Figs. 2, 3 and Supplementary Table 1).

### Structural characterization of the filaments and cylinders

Overall, each cylindrical fiber consists of coaxially stacked double-layered rings ~28 nm in diameter, each containing 26 TIR_MyD88_ subunits per layer in each round (Fig. 3a, b). Each of the double-layered rings consists of two identical rings stacked head-to-head (Fig. 3c, d). To form the cylinder, the double-layered rings are stacked coaxially tail-to-tail. The head-to-head stacking interface (referred to as “the dense interface”) is more densely packed than the tail-to-tail interface (“the sparse interface”); the buried surface areas of the dense and sparse interfaces are ~440 Å^2^ and ~337 Å^2^ per subunit, respectively. Detailed examination of the structure revealed that the subunit arrangement in the filament (i.e., equivalent to the double ring) is partially identical to that in the two-stranded filament of TIR_MyD88_ crystallized with the aid of Mal filaments^17^. However, there are considerable differences between our filaments and the Mal-induced oligomers. In particular, the alignment of the two strands is completely opposite. That is, in the Mal-induced oligomers, two TIR_MyD88_ strands are joined in a parallel orientation, whereas in our filaments, the two strands are in an antiparallel orientation (Fig. 3d and Supplementary Fig. 4a–c). Furthermore, in the Mal-induced oligomers, each subunit in one of the strands is wedged between two adjacent subunits in the other strand, whereas in our cryo-EM structure, one subunit in one strand is in complementary and symmetrical contact with one subunit in the other strand. In a sense, this *antiparallel double-stranded* filament can be considered a linear assembly composed of symmetric head-to-head dimers of TIR_MyD88_ (Fig. 3d and Supplementary Fig. 4a).

**Fig. 4:**
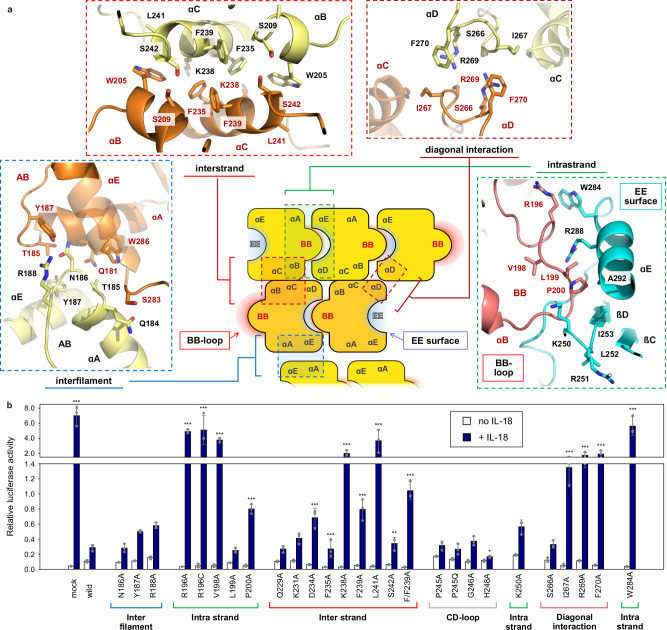
Validation of the double-stranded filament structure. **a** Cartoon showing the arrangement of subunits in the cylindrical fiber and the interfaces of the interstrand (red dotted square), diagonal (dark red dotted square), interfilament (blue dotted square) and intrastrand (green dotted square) interactions depicted with stick models on the ribbon diagrams. **b** NF-κB reporter assay with IL-18-stimulated 293 T cells. Each column shows the NF-κB-driven luciferase activity of stimulated (blue) and unstimulated (white) cells expressing WT TIR_MyD88_ or mutants of TIR_MyD88_. F/F239A: double mutant, F235A and F239A. The data shown are the mean ± SD from triplicate experiments, with individual data points displayed. *p < 0.05, **p < 0.01, ***p < 0.001 (Dunnett’s test vs WT on log_2_-transformed fold-change ( + IL-18/no IL-18) values). Source data are provided as a Source Data file.

For convenience, we can categorize the subunit interactions in the cylinders into three types: “intrastrand,” describing interactions within individual strands; “interstrand”, describing interactions at the dense interface; and “interfilament”, describing interactions at the sparse interface (Fig. 4a). Intrastrand interactions account for 8 ~ 9% (~745 Å^2^) of the total surface area of a single subunit. These interactions are essentially identical to those in the Mal-induced oligomers. Based on previously proposed nomenclature^17^, the BB surface of one subunit binds to the EE surface of the adjacent subunit (Fig. 4a, Supplementary Fig. 2c and Supplementary Table 2), and the protein backbone structure in these regions is similar to that in the Mal-induced oligomers (Supplementary Fig. 4d–i). On the other hand, substantial differences from the Mal-induced oligomers were evident in the interstrand interactions, accounting for ~5% (~440 Å^2^) of the total surface area of a single subunit, in which the αB and αC helices (the BC surface) from two subunits interact with each other (Fig. 4a and Supplementary Fig. 2d). In a sense, the two subunits form a head-to-head dimer with C2 symmetry through the BC surface residues, in which W205, F235, K238, F239, and S242 are positioned symmetrically at the interface to participate in extensive hydrophobic interactions with each other (Fig. 4a and Supplementary Fig. 5a). The resultant hydrophobic core is supported by peripheral hydrogen bonds between W205 and S242 (Supplementary Table 3). The symmetric dimers assemble linearly through intrastrand interactions to form a double-stranded filament, in which diagonal interactions also play a role, with hydrophobic contacts between I267 and F270 and hydrogen bonds between S266 and R269 (Fig. 4a, Supplementary Fig. 2c and Supplementary Table 3). Consequently, the lateral surface of the filament is markedly distinct from that of the Mal-induced oligomers (Supplementary Table 3). A notable difference is the location of the N-termini to which DD_MyD88_ should be connected. In our double-stranded filaments (i.e., the rings), all the N-termini are on one side of the filament, whereas in the Mal-induced oligomers, they are directed toward the opposite sides of the oligomers (Supplementary Fig. 4a, b). DD_MyD88_ forms a helical oligomer that serves as the acceptor site for IRAK4. The subunit arrangement in our double-stranded filaments is expected to facilitate the formation of this oligomer because four TIR_MyD88_ subunits form a parallelogram with all four N-terminal ends facing in one direction (red dotted lines in Fig. 3b–d). This arrangement is suitable for the alignment of four DD_MyD88_ subunits to form a helical oligomer because in the oligomer, the C-terminal ends of the four DD_MyD88_ subunits are in a quasisquare configuration (Supplementary Fig. 6). Thus, the formation of the TIR_MyD88_ filament is expected to promote DD_MyD88_ oligomerization (see the Discussion for further details). In contrast, the parallel arrangement induced by Mal does not impose the spatial constraints required for optimal DD_MyD88_ tetramer formation. Thus, although the parallel mode can cluster DD_MyD88_, it does not facilitate their optimal positioning for tetramerization.

The interfilament interactions (the sparse interface) result in the formation of cylindrical fibers from double-layered rings, in which residues from the end of the αA to the AB loop as well as the N-terminal half of the αE play a major role (Fig. 4a and Supplementary Fig. 2d). This interface buries 3 ~ 4% (~337 Å^2^) of the total surface area of a single subunit. This sparse interaction accounts for the observation that a long incubation time is required for the cylindrical fibers to form. Intriguingly, the interfilament interactions, namely, the formation of tail-to-tail dimers, are not symmetric (Fig. 4a and Supplementary Table 4). For example, the backbone oxygen of Q181 in one subunit forms hydrogen bonds with the NH side chain of N186 in the other subunit, but the opposite does not occur (i.e., no hydrogen bonds are formed between N186 of the former subunit and Q181 of the latter subunit). Additionally, the OH side chain of Y187 in one subunit forms a hydrogen bond with the NH side chain of R188 in the other subunit, but again, no hydrogen bonds are formed between R188 of the former subunit and Y187 of the latter subunit (Supplementary Table 4). In addition, the hydrophobic contacts between the aliphatic regions of the interface residues are asymmetrical. In fact, while four αE residues (K282, S283, W286 and T287) of one subunit are involved in the interface, only two αE residues (S283 and T287) of the other subunit are involved (Supplementary Table 4). Thus, the cylinder has polarity.

### Thicker cylinders are formed by spiral winding of the antiparallel double-stranded filaments

We then analyzed the thicker cylindrical fibers. The obtained structure indicated that the cylinder is formed from a single antiparallel double-stranded filament wound into a spiral (Supplementary Figs. 3c, 7). However, the subunits are arrayed in essentially the same fashion as in the thinner cylinder, and the intra- and interstrand interactions, as well as the interfilament interactions, are well conserved (Supplementary Fig. 7h, i). Therefore, the two types of cylinders, with different widths, are formed from a common double-stranded filament. The difference is whether the filament closes to form a ring or extends infinitely in a spiral, and this comes from the cumulative effects of slight variations in the placement of each subunit (Supplementary Fig. 7f, g, j). The origin of the two distinct types of cylindrical fibers from a common double-stranded filament indicates that this self-assembly mode is an inherent property encoded in the TIR_MyD88_ structure, suggesting its biological significance.

### Functional validation of the filament structure

To verify whether our antiparallel double-stranded filament of TIR_MyD88_ reflects its functional state in cells, we conducted a cell-based assay to assess the dominant-negative inhibitory effect of TIR_MyD88_ on NF-κB activity^14,31,32^. We ectopically expressed either TIR_MyD88_ or its mutants in HEK293T cells and evaluated NF-κB activation induced by IL-18, a member of the IL-1 family. The expression of wild-type TIR_MyD88_ abrogated NF-κB activity (Fig. 4b, wild), indicating that the expression of TIR_MyD88_ inhibited the proper function of endogenous MyD88. Thus, the ectopically expressed TIR_MyD88_, which lacks DD_MyD88_, likely competes for the multivalent TIR-TIR interfaces required for the productive assembly of endogenous MyD88, thereby preventing the formation of functional oligomers competent to recruit IRAK4. Recently, a dominant-negative inhibitory effect exerted by a short splice variant of MyD88 (MyD88S), which lacks the linker region between DD_MyD88_ and TIR_MyD88_, was investigated. The variant was able to interact with full-length MyD88, but functional oligomers did not form in cells^4^. A similar effect may underlie our observations; however, we did not directly test this mechanism, and its operation remains to be demonstrated.

In contrast, in cells expressing one of the TIR_MyD88_ mutants harboring mutations at the intrastrand interface (R196A, R196C, V198A, or W284A), marked recovery of NF-κB activity was observed, revealing the critical importance of these residues in forming co-assemblies with endogenous MyD88 (Fig. 4b). In fact, the importance of these intrastrand residues in both IL-1R^33^ and TLR signaling^17,34^ has been established. Negative stain TEM revealed that TIR_MyD88_ with the W284A mutation (located on the EE surface) completely failed to self-assemble (Supplementary Fig. 8a). Thus, the reduced dominant-negative effects are likely attributable to impaired intrastrand TIR-TIR interactions, which may prevent proper co-assembly with endogenous MyD88. However, despite the complete loss of self-assembly in vitro, the P200A mutant (positioned at the tip of the BB loop) retained some dominant negative effects. Thus, this mutation may have not completely disrupted co-assembly between mutant TIR_MyD88_ and endogenous MyD88 in the cellular context.

We then examined the interstrand residues that are unique to our double-stranded filaments (red and dark red dotted squares in Fig. 4a). When K238, L241, I267, R269, or F270 was replaced by Ala, significant recovery of NF-κB activity was observed, suggesting that these residues were critical for forming co-assemblies with endogenous MyD88 (Fig. 4b). Interestingly, despite the K238A TIR_MyD88_ mutant did not interfere with endogenous MyD88 in the cells, it was able to form small rings by itself in vitro (Supplementary Fig. 8b). The cryo-EM map of the K238A ring shows that the subunit arrangement is different from that of the WT ring and is incompatible with WT TIR_MyD88_ (Supplementary Fig. 8c–e). Thus, the K238A mutant is not expected to stably co-assemble with endogenous MyD88, though it can self-assemble. Mutations at P245, G246 or H248 in the CD loop did not affect NF-κB activity, suggesting that the contribution of these residues to signal transduction is negligible (Fig. 4b). Consistently, these residues were exposed to the solvent in the oligomers, with little or no density map. In contrast, in the Mal-induced oligomers, these residues were buried at the interstrand interface (Supplementary Tables 2,3 and Supplementary Fig. 5c)^17,34^.

Next, we examined the interfilament interface (Fig. 4a, blue dotted square). Replacement of either N186, Y187, or R188 with Ala resulted in only a small reduction in the dominant-negative inhibitory effect, indicating that these residues have limited contributions to forming co-assemblies with endogenous MyD88 in the cells (Fig. 4b). This finding is consistent with a previous report showing that neither N186A nor R188A affected IL-1β-induced NF-κB activity^33^. TEM revealed that TIR_MyD88_ with the Y187A mutation formed rings similar to those formed by WT TIR_MyD88_ but largely failed to form cylindrical fibers (Supplementary Fig. 8a). Thus, although the interfilament interactions are required for forming the cylindrical assembly in vitro, they are dispensable for functional oligomers in cells. Taken together, these data strongly support that the antiparallel double-stranded filament structure represents the functional oligomeric state of TIR_MyD88_ in cells.

### HS-AFM reveals the mode of subunit incorporation during strand elongation

**Fig. 5:**
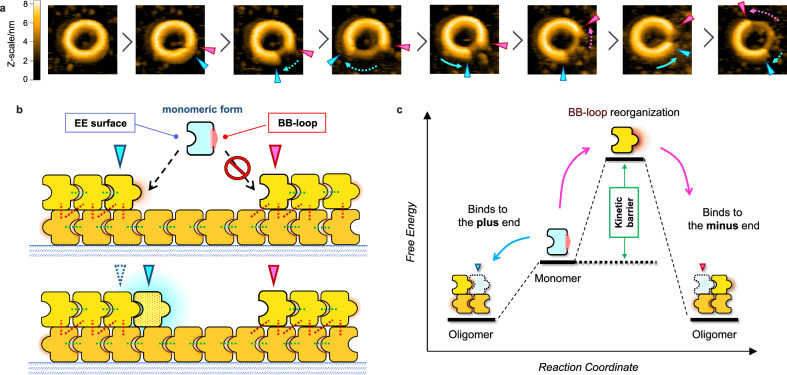
Unidirectional regeneration of the upper ring of TIR_MyD88_. **a** Time series of AFM images showing unidirectional regeneration of the upper ring (Supplementary Movie 1). Scan area: 60 × 60 nm^2^ with 120 × 120 pixels. Scan speed: 600 ms per image. Disintegration of the end denoted by the cyan triangle occurs in a clockwise direction (cyan dotted arrow), and regeneration then occurs in a counterclockwise direction (cyan arrow). Disintegration of the other end, denoted by the pink triangle, occurs in a counterclockwise direction (pink dotted arrow), and regeneration does not occur at this end, indicating the lack of subunit incorporation at this end. **b** Schematic model of upper strand regeneration. The free subunit binds only to the plus end (cyan triangle) of the upper strand where the BB loop is exposed and does not bind to the minus end (pink triangle) where the EE surface is exposed. The convex surface represents the exposed BB loop, whereas the concave surface represents the EE surface. Notably, in the free subunit, the BB loop folds back onto the main body (Supplementary Figs. 4e, f, 5a, b). **c** Proposed free energy diagram illustrating the monomer incorporation into the upper strand shown in (**b**). Binding of the monomer to the minus end requires reorganization of the BB loop, which imposes a kinetic barrier. Representative images from more than three independent experiments with consistent results are shown.

During HS-AFM measurements of TIR_MyD88_ rings, we often observed disintegration and regeneration of the rings. The disintegration was caused partially by the inevitable scanning forces of the AFM cantilever on the specimen. Closer examination of the ring disintegration/regeneration process (Supplementary Movie 1) showed that the upper ring disintegrated while the lower ring remained intact. The upper ring subsequently regenerated on top of the lower ring. The most striking feature was that while disintegration of the upper ring occurred in both directions, regeneration occurred only in a counterclockwise direction (Fig. 5a). In other words, the elongation of the upper strand is unidirectional.

In reference to our cryo-EM structure, regeneration occurs such that the exposed BB loop of the subunit at one end (designated the plus end) is bound by the EE surface of a free subunit. In contrast, the exposed EE surface of the subunit at the other end (minus end) is not bound by the BB loop of a free subunit (Fig. 5b). Structural comparison of TIR_MyD88_ in the rings with monomeric TIR_MyD88_^15^ revealed marked differences in the BB surface. Whereas in the monomers, the BB loop folds onto the body, in the rings, it projects to bind to the EE surface of a neighboring subunit (Supplementary Movie 2, Supplementary Figs. 4e, f, 5a, b). Thus, upon incorporation into a ring, the backbone of the BB loop undergoes a significant reorganization to settle into the intrastrand interface, and some of the side chains even translocate to the interstrand interface, thereby contributing to head-to-head dimerization. In contrast, the changes in the EE surface are subtle (backbone root mean square differences (RMSDs) of 7.9 Å and 0.82 Å for the BB and EE surfaces, respectively; Supplementary Fig. 4e). Based on this structural comparison, we speculate that the structural changes in the BB loop occur in concert with the interstrand interactions of TIR_MyD88_ prior to its binding to the EE surface. In this scenario, at the plus end of the upper strand (cyan triangle in Fig. 5), the BB loop is thought to be already rearranged in a form that facilitates its binding to the EE surface of another subunit, thus promoting subunit incorporation at that site. In contrast, the BB loop of free subunits is not prepared for binding; thus, the EE surface at the minus end may be bound only rarely by free subunits (pink triangle in Fig. 5). Namely, the BB loop rearrangement is thought to be a kinetic barrier for the binding process (Fig. 5c). This difference in reactivity toward free subunits between the two open ends may be responsible for the observed counterclockwise elongation of the upper strand.

Because our observation is limited to the regeneration of the upper rings located on top of the preformed lower rings on the AFM stage, the exact process of double-stranded filament formation in either solution or in cells remains undetermined. However, the data indicate that the structural reorganization of the BB loop in free monomers is a slow process that may limit the establishment of intrastrand interactions. Thus, this step may impose a kinetic barrier to the initiation of TIR_MyD88_ self-assembly, preventing unwanted self-assembly and signaling unless the receptor is activated.

### HS-AFM reveals direct binding between the receptor TIR and the TIR_MyD88_ ring

**Fig. 6:**
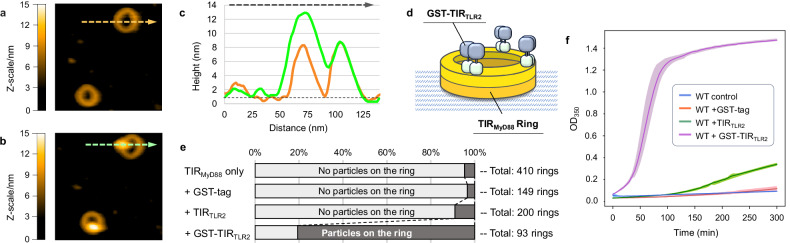
Direct visualization of GST-TIR_TLR2_ binding to TIR_MyD88_ rings. **a** Representative AFM image of preformed TIR_MyD88_ rings. **b** Rings bound by GST-TIR_TLR2_. Scanning area and scan speed in (**a**) and (**b**): 200 × 200 nm^2^ with 120 × 120 pixels and 500 ms per image. **c** Measurements of the cross sections represented by the dashed arrows in the AFM images in (**a**) (orange) and (**b**) (green). **d** Schematic model of the TIR_MyD88_ ring bound by GST-TIR_TLR2_. **e** Frequency of topping events. The dark areas of the bars indicate the percentages of rings with particles among the total number of rings. Rings with particles were frequently observed when GST-TIR_TLR2_ was added, whereas they were much less frequent when either GST or TIR_TLR2_ alone was added. **f** Turbidity assay of TIR_MyD88_ in the presence of GST-TIR_TLR2_, GST, or TIR_TLR2_. The addition of dimeric GST-TIR_TLR2_ markedly accelerated the increase in turbidity, but to a much lesser extent, the addition of monomeric TIR_TLR2_. Data are presented as the mean turbidity of triplicate experiments. Shaded bands indicate the range between minimum and maximum values. Representative images from more than five independent experiments with consistent results are shown. Source data are provided as a Source Data file.

Interactions between IL-1Rs/TLRs and MyD88 are mediated by homotypic interactions of their TIR domains. Since previous reports have demonstrated direct binding between GST-TIR_TLR2_ and TIR_MyD88_^18,35^, we sought to visualize this TIR-TIR interaction using HS-AFM. First, TIR_MyD88_ rings were prepared on the AFM stage (Fig. 6a), and GST-TIR_TLR2_ was added at a final concentration of 5 μM. As shown in Fig. 6b–d and Supplementary Movie 3, topping of the particles on the rings was observed. We determined the frequency of such topping events in the HS-AFM movies recorded in the presence and absence of GST-TIR_TLR2_ as well as following the addition of either GST or TIR_TLR2_ alone (Fig. 6e). The findings demonstrated that these topping events were observed almost exclusively in the presence of GST-TIR_TLR2_. Because GST exists as a dimer, it is assumed that TIR_TLR2_ is forced to dimerize in the corresponding fusion proteins. Thus, these results indicate that only dimeric TIR_TLR2_ binds to TIR_MyD88_ rings.

While TIR_TLR2_ has been reported to form stable homodimers^36,37^ and TLR2 can function as a homodimer under certain conditions^38,39^, TLR2 is thought to function mainly as a heterodimer with TLR1 or TLR6 in vivo. Thus, to better reflect physiological conditions, we further tested another receptor TIR (TIR_TLR5_), which functions exclusively as a homodimer. GST-TIR_TLR5_ bound to preformed rings via the same mode as GST-TIR_TLR2_ (Supplementary Fig. 9), suggesting that the binding mode of these receptor TIRs is common. Importantly, both GST-TIR_TLR2_ and GST-TIR_TLR5_ bound to the upper surface of the rings, indicating that the interfilament surface of TLR_MyD88_ (Fig. 4a) functions as the binding site for the receptor TIRs.

### The self-assembly of TIR_MyD88_ is markedly accelerated by dimeric receptor TIRs

To determine the effect of GST-TIR_TLR2_ on the self-assembly of TIR_MyD88_ in vitro, we conducted a turbidity assay. When the concentration of TIR_MyD88_ was about 190 μM, the turbidity of the solution increased slowly after a lag time (blue line in Fig. 6f). However, a significant change was observed in the presence of a small amount of GST-TIR_TLR2_ (5% of the amount of TIR_MyD88_), with a rapid increase in turbidity observed (purple line in Fig. 6f). Addition of the GST tag alone did not result in this increase in turbidity, demonstrating that the accelerated self-assembly is caused by TIR_TLR2_. Interestingly, only marginal acceleration was observed when untagged TIR_TLR2_ (i.e., without the GST tag) was added (green line in Fig. 6f). TIR_TLR2_ has been reported to form both homodimers and tetramers at high concentrations^36,37^. Given that GST is dimeric, TIR_TLR2_ is likely dimerized in its GST fusion protein (Supplementary Fig. 9d). Thus, these data suggest that dimerization (or at least high local concentration) of TIR_TLR2_ plays an important role in the accelerated self-assembly of TIR_MyD88_. In addition, the amount of GST-TIR_TLR2_ required for the promotion of self-assembly was only 5% that of TIR_MyD88_, strongly suggesting that GST-TIR_TLR2_ is required only at the initial stage of assembly. Most likely, GST-TIR_TLR2_ assembles multiple TIR_MyD88_ subunits at one location to promote the nucleation process. After nucleation, TIR_MyD88_ self-assembly can occur rapidly, as previously hypothesized^10,13^. Thus, these data demonstrate that dimeric receptor TIRs initiate the self-assembly of TIR_MyD88_ by promoting nucleation and that the effect of the dimers is substantially greater than that of the monomers.

## Discussion

### Tetramerization of TIR_MyD88_ is a plausible key step for its self-assembly

**Fig. 7:**
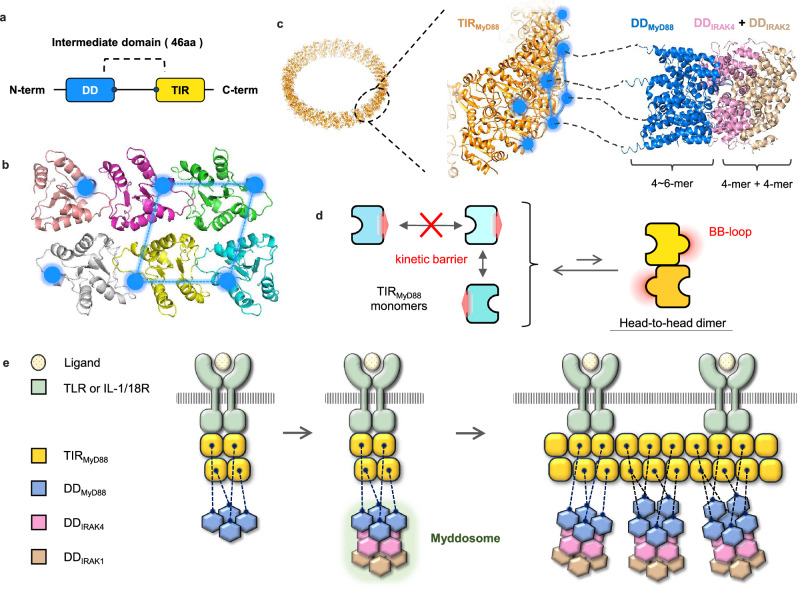
Schematic model of receptor-triggered oligomerization of MyD88. **a**,** b** Domain structure of MyD88 (**a**) and subunit arrangement in filaments (**b**). The filled cyan circles indicate the positions of the N-termini of TIR_MyD88_, to which DD_MyD88_ subunits are connected via linker residues. A parallelogram connecting the N-termini is shown in cyan. **c** Putative spatial relationship between the ring and the DD oligomer, in which TIR_MyD88_ (orange) and DD_MyD88_ (blue) are connected by the intermediate domain (dotted lines). DD_IRAK4_ (pink) and DD_IRAK2_ (light brown) are also shown. **d** Structural reorganization of the BB loop imposes a kinetic barrier for the intrastrand interactions, whereas head-to-head dimerization of TIR_MyD88_ induces rearrangement of the BB loop (red). **e** Schematic of receptor-triggered Myddosome formation and its clustering.

The parallelogram arrangement of four neighboring TIR_MyD88_ subunits is geometrically suitable for promoting the oligomerization of its N-terminal DD_MyD88_ (Fig. 3b–d), because all the N-termini of TIR_MyD88_ protrude to one side in this arrangement (outer surface of the rings, Fig. 7a–c and Supplementary Fig. 4a). In DD_MyD88_ tetramers, the four subunits are arranged in a quasi-square configuration, and their C-termini protrude to one side (Supplementary Fig. 6). Therefore, by facing the N-terminal side of TIR_MyD88_ toward the C-terminal side of DD_MyD88_, a complete arrangement of the full-length MyD88 tetramer was envisaged (Fig. 7c). Because of the intermediate domain^4,8^, the relative positions of TIR_MyD88_ and DD_MyD88_ were not fixed. However, on average, the tetramerization of TIR_MyD88_ brings the four N-terminal DD_MyD88_ subunits into proximity, with a favorable orientation that promotes DD_MyD88_ tetramerization. Thus, the self-assembly of TIR_MyD88_ in an antiparallel arrangement may promote the self-assembly of DD_MyD88_, which in turn initiates the recruitment of IRAK family members via DD-DD interactions. This feature is reminiscent of a mechanism called signaling by cooperative assembly formation, which is frequently found in the innate immune and cell death signaling pathways^40^. In contrast, the Mal-dependent parallel arrangement^17^ of TIR_MyD88_ oligomers lacks the spatial constraints that arrange DD_MyD88_ in optimal positions. This arrangement is expected to drive Myddosome formation less efficiently than by the antiparallel mode (see Results). Nevertheless, the strong clustering capacity of Mal in cells might offset this reduced efficiency, although this remains speculative and requires further experimental testing.

The tetrameric unit of TIR_MyD88_ is stabilized by inter- and intrastrand interactions, which are anticipated to work cooperatively and impart much greater stability than the trimeric or dimeric units. However, the establishment of intrastrand interactions requires a drastic rearrangement of the BB loop and its surrounding residues (Supplementary Fig. 5 and Supplementary Movie 2). Given the polarity of strand elongation observed via AFM (Fig. 5), this rearrangement may impose a kinetic barrier that hampers intrastrand interactions between free monomers. At physiological concentrations, this barrier is expected to prevent uncontrolled and unwanted intracellular self-assembly of TIR_MyD88_ (Fig. 7d). However, after the formation of a stable tetramer owing to interactions with dimerized receptors, the incorporation of free subunits into the tetramer is expected to be markedly promoted because the tetramer can act as a stable platform containing BB loops suitable for binding, leading to rapid elongation of the antiparallel double-stranded filament.

In contrast, the interstrand interface requires less structural reorganization for self-assembly, implying that head-to-head dimers form more readily, although their lifetimes may be shorter (Fig. 7d). This transient dimerization can induce BB loop rearrangement. Hence, in cells, these dimers may associate via interactions with the dimerized receptor TIRs to form tetramers. In addition, TIR_MyD88_ subunits can bind head-to-head dimers to core tetramers (and preformed filaments) during further filament elongation. Although these possibilities await further investigation, the locations of oncogenic mutations that aberrantly facilitate MyD88 oligomerization support this hypothesis. Most of these mutations were in the BB loop and head-to-head dimer interface (the interstrand interface, Supplementary Movie 2, and Supplementary Fig. 5a). If a mutation lowers the kinetic barrier and stabilizes the head-to-head dimer of TIR_MyD88_, core tetramers and double-stranded filaments can readily form, even in the absence of dimerized receptors, leading to constitutive and pathogenic signaling.

Based on these results, we propose that TIR_MyD88_ tetramerization functions as a checkpoint for signal transduction. Atomic force microscopy measurements revealed that both GST-TIR_TLR2_ and GST-TIR_TLR5_ specifically bound to the upper surface of the rings (Fig. 6 and Supplementary Fig. 9). This spatial arrangement suggests that TIR_MyD88_ tetramers are formed below the dimeric receptor TIR (nucleation), from which filament elongation is initiated. It was previously demonstrated that upon stimulation of IL-1Rs in EL4.NOB cells, GFP-fused MyD88 assembled at receptor sites and formed clusters. The number of GFP-MyD88 molecules in the clusters was determined based on fluorescence intensity measurements^13^. Small oligomers (2–3 MyD88 monomers) have been reported to be unstable with short lifetimes, whereas larger oligomers have longer lifetimes and are more likely to coassemble with IRAK4. Thus, the authors proposed that MyD88 oligomer size serves as a “physical threshold” for signal initiation. This hypothesis is consistent with the insights gained from our findings, which indicate that TIR_MyD88_ tetramerization is a critical step in the elongation of functional filaments (Fig. 7c). Taken together, we propose a model for the functional self-assembly of MyD88 triggered by IL-1Rs and a subset of TLRs that does not require Mal (Fig. 7e). In this model, TIR_MyD88_ is first released from the autoinhibited state^5^ and transiently forms symmetric head-to-head dimers via interstrand interactions (Fig. 7d). After the membrane receptors dimerize, their TIR domains directly interact to assemble TIR_MyD88_, which promotes TIR_MyD88_ tetramerization (nucleation step; Fig. 7e, left). The stable tetramer functions as a platform for subunit incorporation, triggering filament elongation. This, in turn, promotes the oligomerization of DD_MyD88_ along the filament, ultimately leading to Myddosome formation at multiple sites on the filament (Fig. 7e). Using this model, the formation of multi-Myddosome clusters is also feasible^41^.

### Functional form of the double-stranded filaments of TIR_MyD88_

Because the cylindrical fibers of TIR_MyD88_ take much longer to form than do the rings (Fig. 1a) and the interfilament mutants do not significantly affect the dominant negative inhibitory effect (Fig. 4b), these cylindrical assemblies may represent an in vitro artifact. In addition, the average number of GFP-MyD88 molecules that accumulated at IL-1Rs recruiting IRAK4 was previously found to be 11 on average^13^, whereas the number of subunits in the double-layered ring of TIR_MyD88_ was at least 52 (Fig. 3). Therefore, relatively short, free double-stranded filaments are thought to constitute the functional oligomeric state of TIR_MyD88_. The reported stoichiometries of DD_MyD88_ and DD_IRAK4_ in DD complexes vary, with ratios of 6:4, 7:4, and 8:4^42,43^. Our double-stranded filament of TIR_MyD88_ is compatible with any of these stoichiometries because DD_MyD88_ subunits, which are connected to TIR_MyD88_ subunits outside the parallelogram formed by the four TIR_MyD88_ subunits, can also participate in DD-DD oligomerization (Fig. 7c). The incorporation of two, three, or four additional DD_MyD88_ subunits, corresponding to the aforementioned stoichiometries, further stabilized the DD_MyD88_ oligomer, resulting in a more robust platform for IRAK4 recruitment.

### An evolutionarily conserved self-assembly mode of the TIR domain

Recently, self-assembled structures of the TIR domains of plant and bacterial proteins have been reported^44–48^. The filament of TIR-STING, an antiphage effector protein of *Sphingobacterium faecium*, is noteworthy^44^, as the tetrameric subunit arrangement in this filament is approximately the same as that in the double-stranded filament of TIR_MyD88_ despite its evolutionarily distant relationship (Supplementary Fig. 10a, b). Formation of TIR tetramers has also been reported for other TIR-containing proteins. Examples include hSARM1^46^, ROQ1 (a protein related to plant immunity)^45^, and RPP1^47^ (Supplementary Fig. 10c–h), although their subunit arrangements differ from those of the TIR_MyD88_ assemblies. Another noteworthy feature of the TIR domains of these proteins is that they commonly utilize a surface that we defined as the interfilament surface for tetramerization (Supplementary Fig. 10c–e). In this regard, our AFM measurements identified the interfilament surface as the binding site for dimeric receptor TIRs (Fig. 6 and Supplementary Fig. 9). Therefore, it may be valuable to focus on the interfilament surface to determine the mode of interaction between the receptor and MyD88.

In summary, we determined the structure of TIR_MyD88_ filaments using cryo-EM. This architecture provides a geometric explanation for the regulated recruitment of downstream IRAKs via antiparallel double-stranded filament formation, as well as insights into disease mutations. Our HS-AFM and turbidity assay results greatly advance our mechanistic understanding of receptor-triggered oligomerization of MyD88. The structure and molecular scheme proposed herein will prompt further mechanistic analyses of this key adaptor protein and increase our understanding of Myddosome function.

## Methods

### Construction of expression vectors

The coding region of human TIR_MyD88_ (amino acids 153–296; numbering according to RefSeq NP_002459.3) was cloned into the pET-28a vector (Novagen), which was engineered with a 6×His-tagged streptococcal protein GB1 domain (GB1) for protein expression and purification. For the luciferase activity assay, TIR_MyD88_ (residues 148–296; numbering per NP_002459.3) with an N-terminus myc epitope was also cloned into the pcDNA3.1+ vector (Invitrogen). The derivatives, including alanine-substituted derivatives, were prepared by site-directed mutagenesis. The *TLR2* gene encoding the TIR domain (amino acid residues 633–784) and the *TLR5* gene encoding the TIR domain (amino acid residues 685–858) were each cloned separately into a pGEX-6P-1 vector^35^. IL-18Rβ construct tagged at the C-terminus with an AU1-epitope was also cloned into pcDNA3.1 + . The NF-κB luciferase reporter vector (pGL4.32-luc2P/NF-kappaB-RE/Hygro) and the Renilla luciferase reporter vector (pGL4.74-hRluc/TK) were purchased from Promega.

### Protein expression and purification

For experimental samples of TIR_MyD88_, the expression vector for the His-GB1 tag fusion TIR_MyD88_ was introduced into *Escherichia coli* BL21(DE3) cells. The cells transformed with the His-GB1-tagged fusion TIR_MyD88_ vector were grown in LB medium. The His-GB1-tagged fusion TIR_MyD88_ was first purified with His-tag affinity column chromatography (cOmplete™ His-Tag Purification Resin: Roche). After removal of the His-GB1 tag with GST-HRV3C, the protein was further purified with cation exchange chromatography (HiTrap™ SP HP: Cytiva) and size-exclusion chromatography (HiLoad® 16/600 Superdex® 75 pg: Cytiva).

For experimental samples of GST-TIR_TLR2/TLR5_, the expression vectors were introduced into *Escherichia coli* BL-21 (DE3) (Novagen). The expressed GST fusion proteins were purified by Glutathione Sepharose™ 4 FF (Cytiva) affinity chromatography and size-exclusion chromatography (HiLoad® 16/600 Superdex® 75 pg: Cytiva)^35^. For preparing TIR_TLR2_, before size-exclusion chromatography, the GST-tag was removed by digestion with GST-HRV3C followed by anion exchange chromatography (HiTrap^TM^ Q HP: Cytiva).

### Dominant negative assay with NF-κB reporter gene activity

HEK293T cells (RIKEN BRC) were cultured in Dulbecco’s Modified Eagle Medium (high glucose-containing D-MEM, Invitrogen) supplemented with 10% heat-inactivated fetal bovine serum (SIGMA-ALDRICH), penicillin (100 U/mL), and streptomycin (100 µg/mL). All cells were incubated at 37 °C in a humidified atmosphere of 5% CO_2_. For the reporter gene assays, HEK293T cells were transfected with 50 ng per well of pcDNA3.1+ mock vector or pcDNA3.1+ myc-TIR_MyD88_ (wild-type or mutants) in 96-well plates using Lipofectamine 2000 (Invitrogen) according to the manufacturer’s instructions. The NF-κB luciferase reporter, Renilla luciferase reporter vectors and pcDNA3.1 + IL-18Rβ were co-transfected. After transfection, cells were incubated for 24 h, then stimulated with recombinant human IL-18 (20 ng/mL) for 6 h. Luciferase reporter activity was analyzed using the Dual-Luciferase Reporter Assay System (Promega) as per the protocols. The fold change (IL-18/no IL-18) was calculated for each replicate. The resulting values were log_2_-transformed for statistical analysis. One-way ANOVA was performed to assess overall group differences, followed by Dunnett’s multiple comparisons test to compare each group with the WT control. Statistical significance was defined as follows: *p < 0.05, **p < 0.01, and ***p < 0.001.

### Turbidity assay

Self-assembly of TIR_MyD88_ was monitored at 37 °C in 20 mM HEPES pH 7.0, 50 mM NaCl, 5 mM DTT by the change in turbidity (optical density at 350 nm) in a UV-Star® Microplate (Greiner Bio-One). The TIR_MyD88_ samples without or with TIR_TLR2/5_ were incubated by shaking at 108 rpm for 5 h with a SPARK® multimode microplate reader (TECAN), attached with a Humidity cassette to keep high humidity around the sample.

### Negative-stain Transmission electron microscopy

TEM images were obtained using a JEM-1400Flash (JEOL) operating at an accelerating voltage 120 kV. To observe the time course of self-assembly of TIR_MyD88_, TIR_MyD88_ solution (10–300 μM) was incubated for 1 h to 3 days at 4 °C or 37 °C. All the samples were left intact or diluted 10-fold in 20 mM HEPES pH 7.0, 50 mM NaCl, 5 mM DTT, loaded onto a carbon grid, and stained with 1% uranyl acetate.

### High-speed AFM imaging

A high-speed atomic force microscope (HS-AFM) was equipped with a small cantilever (BL-AC10-DS-A2 (Olympus): spring constant, k = 0.1 N/m, resonance frequency, f = 400 ~ 500 kHz in water) and was operated in tapping mode at room temperature^26,49,50^. An amorphous carbon tip was grown on the top of each cantilever by electron-beam deposition with a scanning electron microscope (ERA-8000FE (Elionix)). The free oscillation amplitude was 1.4 ~ 2 nm, and the typical set-point amplitude was 85% of the free oscillation amplitude. The imaging rate, scan size, and feedback parameters were optimized to enable visualization using a minimum tip force. A mica disk (1.5 mm in diameter) fixed by epoxy glue on a glass rod (1.5 mm in diameter and 2 mm in height) was used as a sample stage^26^. After the sample stage was fixed by nail polish on the z-piezo of the HS-AFM scanner, the mica was freshly cleaved. Then, 2 μL of 10–200 μM TIR_MyD88_ was adsorbed on the substrates in AFM sample buffer containing 20 mM HEPES pH 7.0, 50 mM NaCl, and 10 mM DTT. After 10 min, the sample was rinsed with 20 μL of the sample buffer twice. The sample stage was filled with 63 μL of the sample buffer before starting measurement, and 7 μL of 50 μM GST-TIR_TLR2_, 50 μM TIR_TLR2_, 50 μM GST-TIR_TLR5_ or 50 μM GST was added to the sample stage while scanning.

The force applied between the AFM tip and the sample, which is estimated to be approximately 20–30 pN^30^, may deform the protein structures to some extent. However, the deformation of a typical globular protein owing to this force is estimated to be, at most, a few percent of the protein height, which is smaller than the measurement error of HS-AFM. The AFM height values may contain such distortions; however, no corrections were made in the present study. Of note, the height of the TIR_MyD88_ rings from HS-AFM imaging was almost identical to that from the cryo-EM.

### Processing of HS-AFM data

The HS-AFM image sequences were processed using Kodec 4.5.7.25 developed by the Kanazawa University WPI Nano Life Science Institute (WPI-NanoLSI)^51^. The background height level was adjusted to zero after background leveling in all images. After taking images, we tracked a target molecule using two-dimensional (2D) correlation analysis to compensate for the slow drift of the sample stage position in the x- and y-directions. A 3 × 3 pixel-average filter was applied to each tracked image to reduce noise^52^.

### Cryo-EM sample preparation and data collection

200 μM TIR_MyD88_ in 20 mM HEPES pH 7.0, 50 mM NaCl, 10 mM DTT was incubated for 3 days at 30 °C and 2.5 μL of the solution was applied onto a Quantifoil R 1.2/1.3 200 mesh Cu grid (Quantifoil) and plunge-frozen in liquid ethane by a Vitrobot Mark IV (FEI). The grids were glow-discharged with a 20 mA current for 30 s just before sample application. The parameters for plunge-freezing were as follows: blot time 2.5 s, humidity 100%, temperature 4 °C. After plunge-freezing, residual liquid ethane on the grid was thoroughly blotted off with filter paper and the grid were stored in liquid nitrogen.

Data acquisition was done by the CRYO ARM 300 (JEOL) operated at 300 kV installed with SerialEM^53^ for automatic data collection and YoneoLocr^54^ for the hole alignment at The University of Osaka. The Ω-type in-column energy filter was operated with a slit width of 20 eV for zero-loss imaging. The imaging parameters were: nominal magnification 50,000, defocus −0.5– −2.0 µm, total dose 40 e^−^/Å^2^, exposure time 3.2 s, one image acquisition per a hole. Images were recorded with a K3 direct electron detector (Gatan) in CDS mode at a pixel size of 1.01 Å/pixel and each movie was fractionated into 40 frames.

### Cryo-EM image processing and 3D map reconstruction

A total of 5325 images were collected. Image processing was performed with RELION 3.1 software^55,56^. After motion correction with MotionCorr^57^ and CTF estimation with Gctf^58^, 4930 images were selected for further image processing. The thinner cylindrical filaments were manually picked with e2helixboxer^59^, after which 60,578 particles were extracted as overlapping boxes of 500 × 500 pixels with a step size of 66 Å along a filament. After 2D classification, 26,680 boxes were selected. Initial 3D models for searching a symmetry of the structure were created as assumed the circular symmetric bilayer using SPIDER^60^, determined of C26 symmetry in 3D refinement. 3D reconstruction and refinement was performed with a helical refinement in Cryosparc (v3.2.0) software^61^. Helical symmetry was determined to –9.2° twist/66.6 Å rise along the left-handed helix. Local and Global CTF refinement were performed. The final resolution reached 3.3 Å (Supplementary Figs. 2b, 3b and Supplementary Table 1). The structure determination of the thicker cylindrical fibers was conducted in a manner similar to the thinner ones described above. However, particle picking was performed using the filament tracer tool in Cryosparc, after which fibers of uniform diameter were manually selected and processed (Supplementary Fig. 3a).

### Cryo-EM model building, refinement, and model analysis

The initial atomic model of TIR_MyD88_ were prepared from an atomic model of the crystal structure of the oligomeric state of the TIR_MyD88_ (PDB 7BEQ)^17^. The model was fitted into the EM map using the ‘fit in map’ function of UCSF Chimera program^62^ and was iteratively refined using COOT^63^. Each of the final models was subjected to real-space refinement in PHENIX^64^. PyMOL (version 2.2.3 Schrödinger, LLC), UCSF Chimera^62^, UCSF ChimeraX^65^, and PISA^66^ were used to analyze and visualize the structures and to create the molecular graphics. The RMSD of the residues in the intrastrand interface was calculated using PyMOL based on the protein backbone atoms. The residues of the BB surface that interact with the EE surface are P169, I172, Q173, Q176, I169, and S194-I207. The residues of the EE surface that interact with the BB surface are K250, L252-P254, R269-C274, N278, C280, T281, W284, R288, L289, K291, A292, and L295.

### Reporting summary

Further information on research design is available in the Nature Portfolio Reporting Summary linked to this article.

## Supplementary information

Supplementary Information

Description of Additional Supplementary Files

Supplementary Movie 1

Supplementary Movie 2

Supplementary Movie 3

Reporting Summary

Transparent Peer Review file

## Source data

Source Data

## Data Availability

All data relevant to the conclusions of this manuscript are included in the text, the supplementary information, or the source data file. The cryo-EM density maps generated in this study have been deposited in the Electron Microscopy Data Bank (EMDB) under accession codes EMD-39676 (stacked rings) and EMD-37355 (helical cylinder). The atomic coordinates have been deposited in the Protein Data Bank (PDB) under accession codes 8YYM (stacked rings) and 8W8M (helical cylinder). Source data are provided with this paper. All other data are available from the corresponding author upon request. Source data are provided with this paper.
