# Supplementary material for: Structural Mechanism of Receptor-Triggered MyD88 Oligomeric Assembly in Innate Immune Signaling
Source: Nat Commun. 2026 Apr 17;17:5364. doi: 10.1038/s41467-026-71836-8 (PMC13276377; doi:10.1038/s41467-026-71836-8)
Supplement: Supplementary file 1 — Supplementary Information [file 41467_2026_71836_MOESM1_ESM.pdf]

Supplementary Information for

Structural Mechanism of Receptor-Triggered MyD88 Oligomeric Assembly in Innate Immune Signaling

Kazuki Kasai[1], Kayo Imamura[1], Masatoshi Uno[1], Shiho Nukui[1], Naotaka Sekiyama[1], Tomoko Miyata[2,3], Fumiaki Makino[2,3,4], Ryusei Yamada[5], Yoshiki Takahashi[5], Noriyuki Kodera[6], Keiichi Namba[2,3], Hidenori Ohnishi[7,8,9], Akihiro Narita[10], Hiroki Konno[5,6], Hidehito Tochio[1]*

*Correspondence: tochio@mb.biophys.kyoto-u.ac.jp

This PDF file includes:

Supplementary Figures 1‐10

Supplementary Tables 1‐4

Supplementary Figures

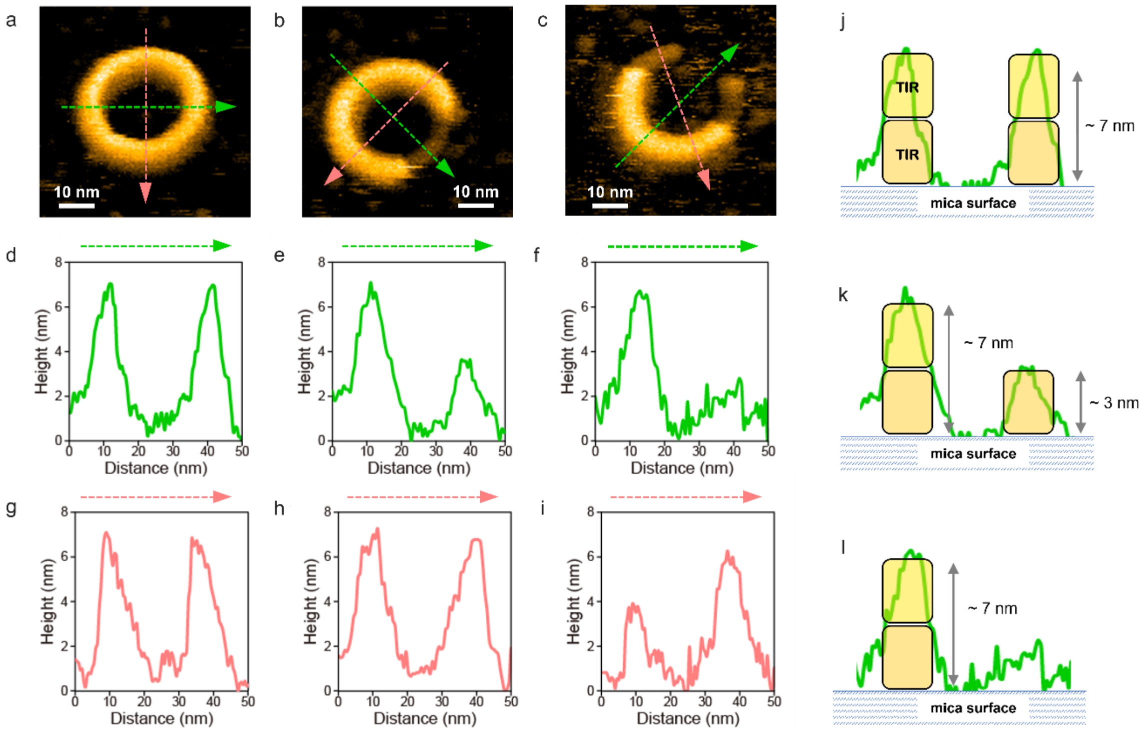

Supplementary Fig. 1: The TIR_{MyD88} ring is double-layered. Related to Fig. 2

(a-c) Representative AFM images of the rings in the disintegration process. Each image was extracted at 13.84 s (a), 17.24 s (b) and 49.40 s (c) in Supplementary Movie 1. Scan area: 60 × 60 nm^2 with 120 × 120 pixels, Z-scale, 8.4 nm. Scan speed: 600 ms per image. (d-i) Sectional views of the green and pink arrows in the AFM images in (a), (b) and (c), respectively. The diameter of the rings was approximately 29 nm, and the height of the double-layered rings was approximately 6.5 nm. In the partially lacking ring, the heights of lacking regions were approximately 3.2 nm (e, i) or less than 2 nm (f). (j-l) Schematic representations of subunit arrangements in (d), (e), and (f), respectively. Representative images from at least three independent experiments with similar results are shown.

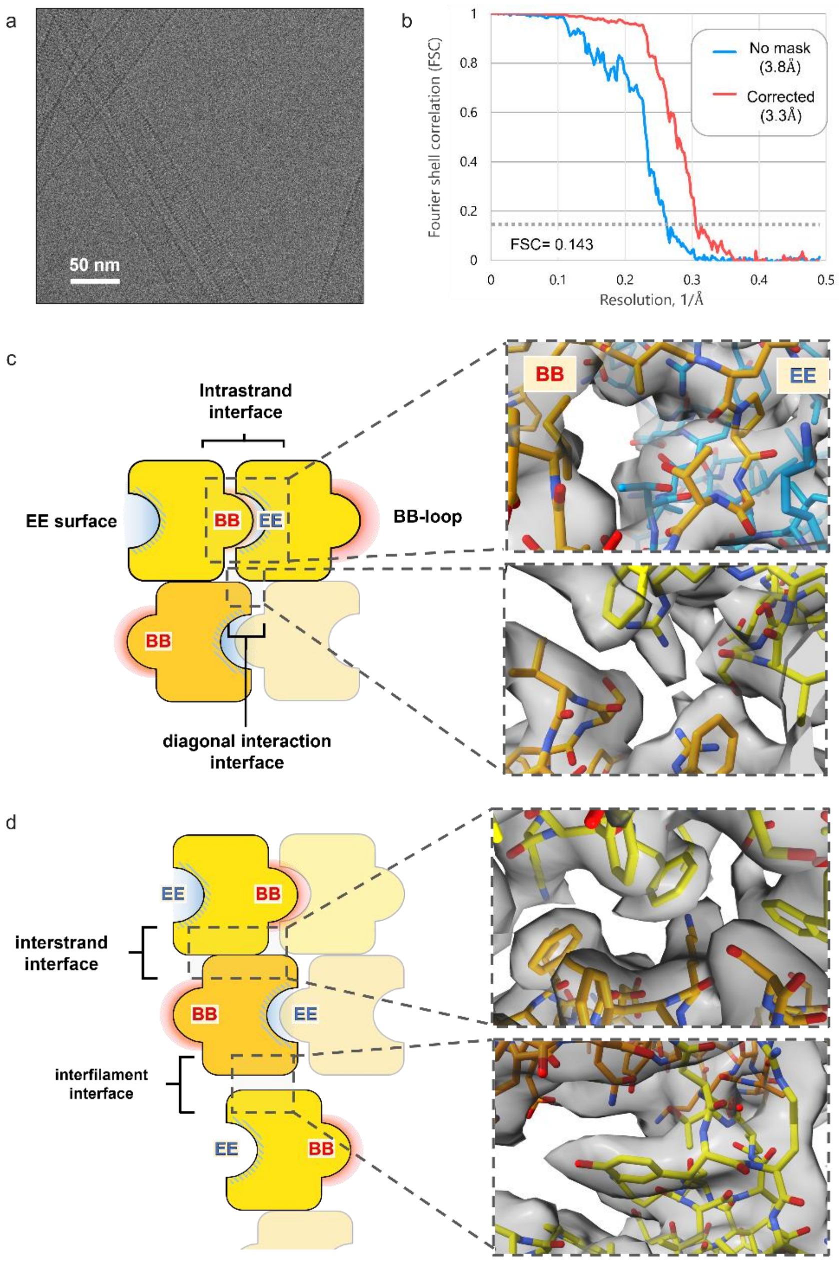

Supplementary Fig. 2: Cryo-EM imaging and the evaluation of the density map. Related to Fig. 3 and Fig. 4a

(a) A typical motion-corrected cryo-EM micrograph of the cylindrical fibers formed by stacked rings. (b) Fourier shell correlations (FSCs) for evaluating the resolution calculated by CryoSPARC v3.2.0 [1]. FSCs between two 3D structures from each half of the dataset with or without masking are presented. Based on the golden standard criterion, the resolution is 3.3 Å with a threshold of 0.143. (c, d) Magnified views of the cryo-EM map around interaction interfaces. (c) External view of the cylindrical fiber, focusing on the interstrand interface (BB loop (orange stick) and EE surface (blue stick), which consists of the βD-strand, βE-strand, and αE-helix) and the diagonal interaction interface (αD helices).

(d) External view of the cylindrical fiber, focusing on the interstrand interface, which consists of αB and αC helices, and the interfilament interface, which consists of αA, AB loop, and αE.

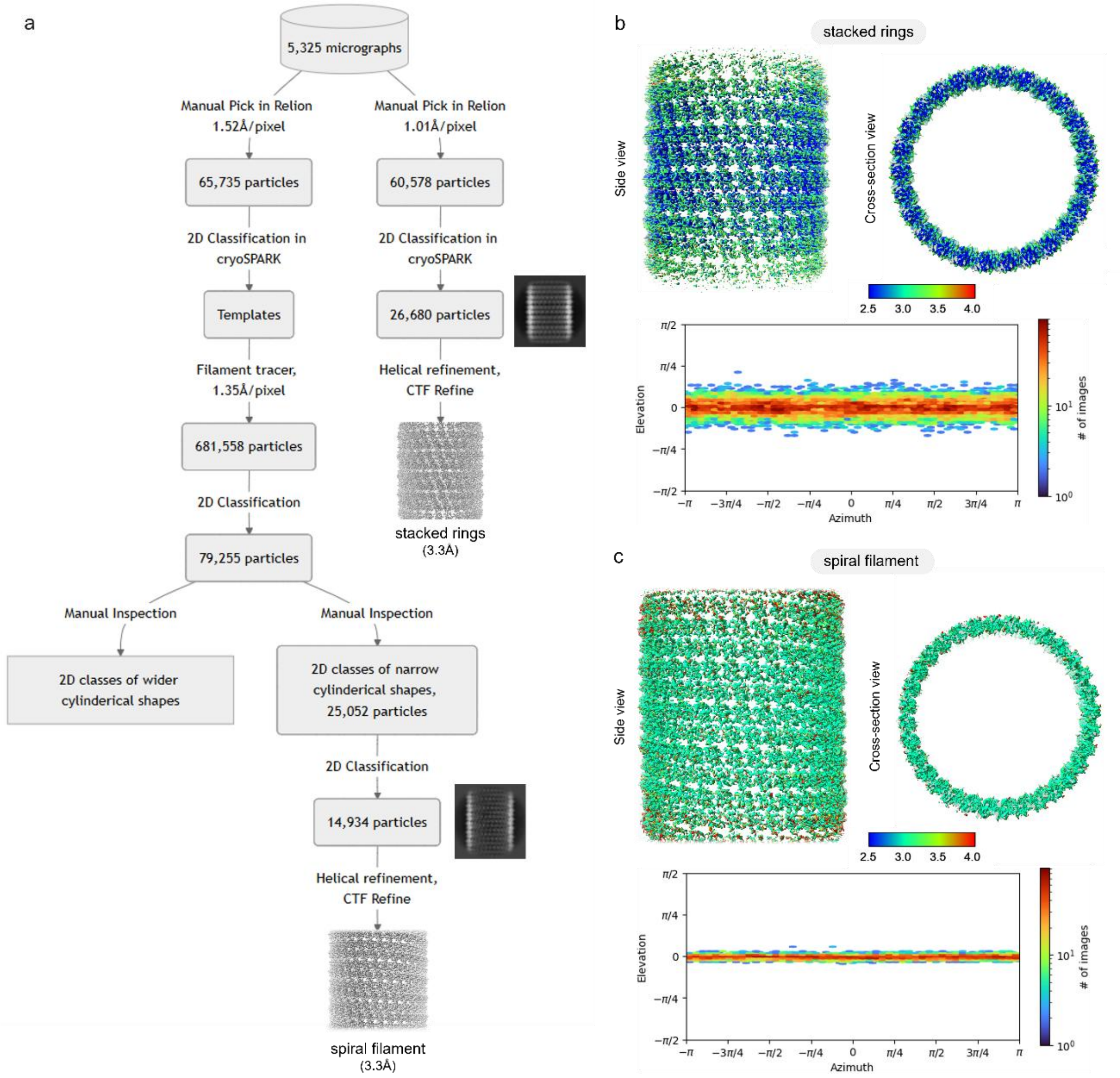

Supplementary Fig. 3: Cryo-EM data processing flowchart of the cylindrical fibers of TIR_{MyD88}
(a) Cryo-EM data processing flowchart. (b, c) Surface representations of the stacked rings (b) and spiral filament (c) colored by local resolution, along with the angular distributions of particles used for the final density map. Local resolution was estimated using ResMap [2].

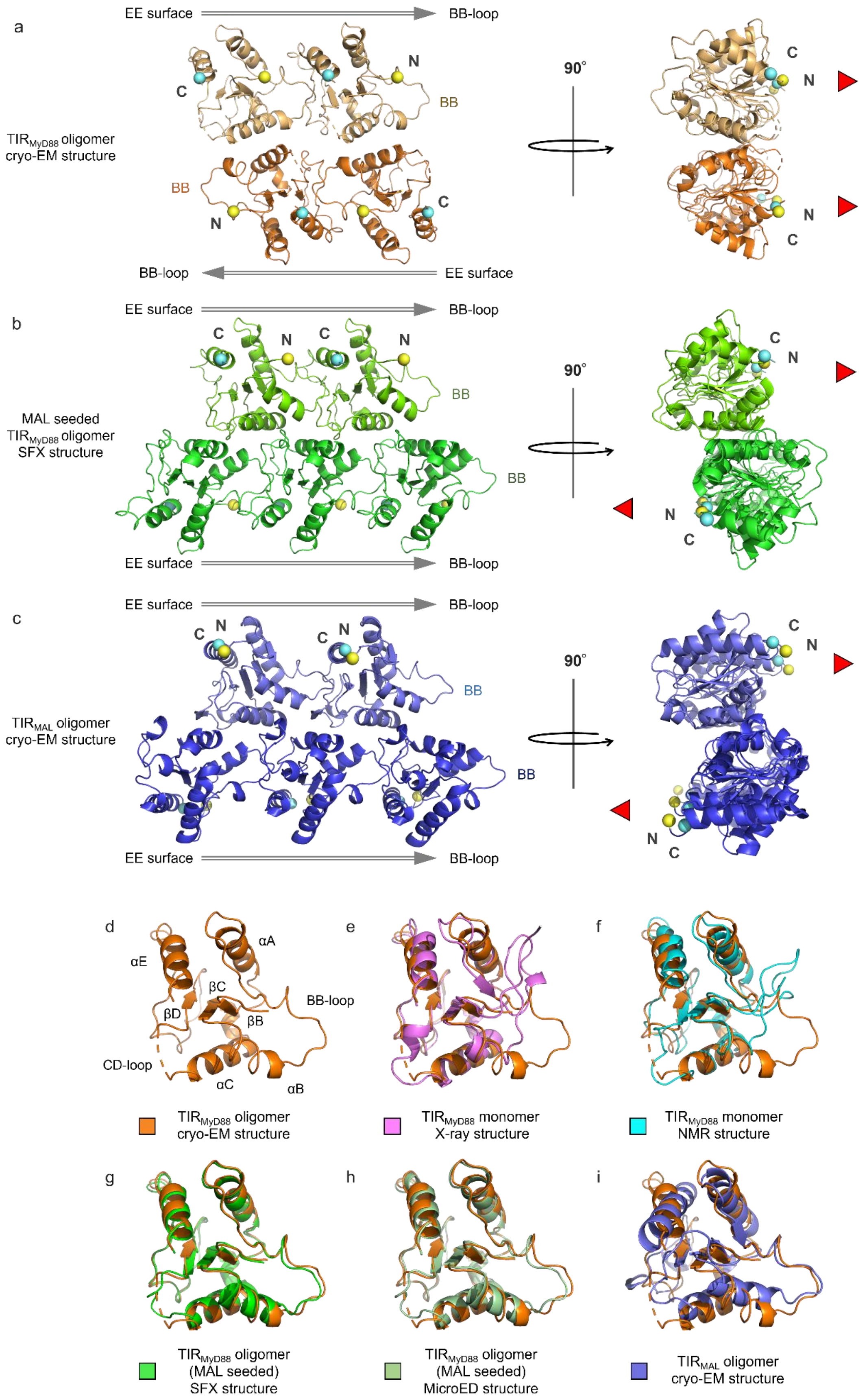

a
EE surface
BB-loop
TIR$_{MyD88}$ oligomer cryo-EM structure
C
N
BB
BB
N
C
BB-loop
EE surface
90°
C
N
N
C
b
EE surface
BB-loop
MAL seeded TIR$_{MyD88}$ oligomer SFX structure
C
N
C
N
BB
BB
EE surface
BB-loop
90°
C
N
N
C
c
EE surface
BB-loop
TIR$_{MAL}$ oligomer cryo-EM structure
C
N
C
N
BB
BB
EE surface
BB-loop
90°
C
N
N
C
d
αA
αE
βC
βD
βB
BB-loop
CD-loop
αC
αB
TIR$_{MyD88}$ oligomer cryo-EM structure
e
TIR$_{MyD88}$ monomer X-ray structure
f
TIR$_{MyD88}$ monomer NMR structure
g
TIR$_{MyD88}$ oligomer (MAL seeded) SFX structure
h
TIR$_{MyD88}$ oligomer (MAL seeded) MicroED structure
i
TIR$_{MAL}$ oligomer cryo-EM structure

Supplementary Fig. 4: Structural comparison of TIR_{MyD88} in various states. Related to Fig. 3

(a-c) The cryo-EM structure in this work (a) and Mal-induced crystal structure (b) [3] of the TIR_{MyD88} oligomer are of a double-stranded linear assembly. In both cases, each strand consists of tandemly arrayed subunits that associate via the intrastrand interactions. However, the alignment of the two strands is completely opposite. In the Mal-induced oligomer, two TIR_{MyD88} strands are joined in a *parallel* orientation (b) identical to that of the Mal fibril (c) [4]. In contrast, in the cryo-EM structure, the two strands are in an *antiparallel* orientation (a). Additionally, in the Mal-induced oligomers, each subunit in one of the strands is wedged between two adjacent subunits in the other strand, whereas in the cryo-EM structure, one subunit in one strand is in complementary and symmetrical contact with one subunit in the other strand. As a result, in (a), all the N-termini, where DD_{MyD88} connects, are located on one side as indicated by the red triangles. In contrast, in (b) and (c), the N-termini project into two opposite sides of the filament as indicated by the red triangles. The N- and C-termini are depicted in yellow and cyan spheres, respectively.

(d-i) Structural comparison of TIR_{MyD88} determined in this study (in orange) with structures from other studies. The structure of the BB loop is significantly different from the monomeric states ((e) PDB ID: 4EO7 in pink and (f) 2Z5V in cyan) [5,6], though similar to other oligomeric states of TIR_{MyD88} ((g) PDB ID: 7BER in green and (h) 7BEQ in pale green) and TIR_{Mal} ((i) PDB ID: 5UZB (purple)) [3,4]. In (e), root mean square differences (RMSDs) for the BB and EE surfaces aligned based on the protein backbone were 7.9 Å and 0.82 Å, respectively.

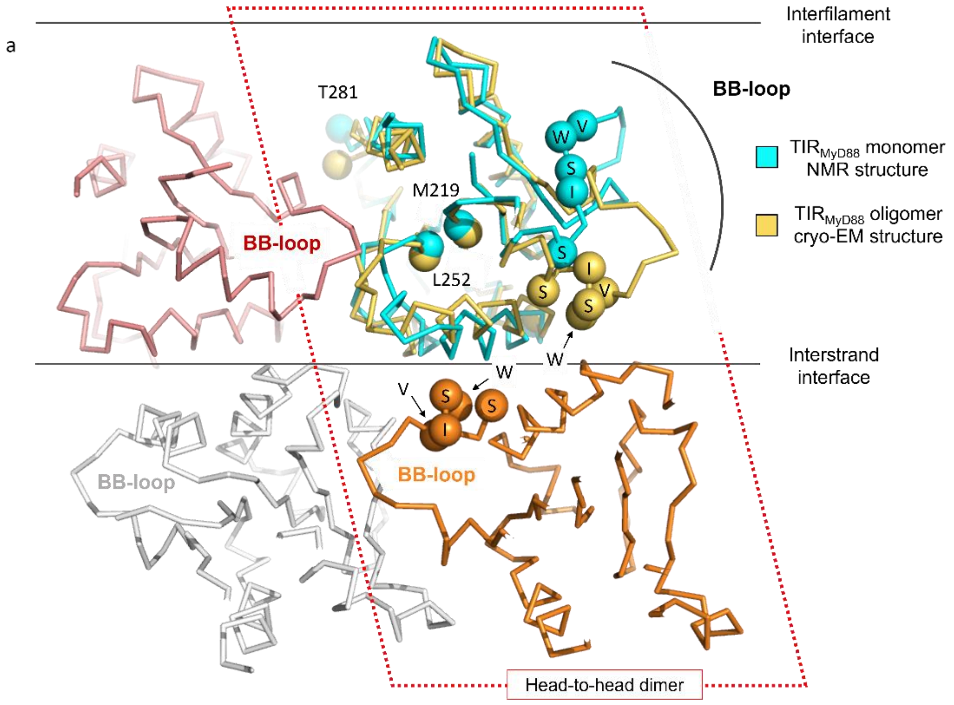

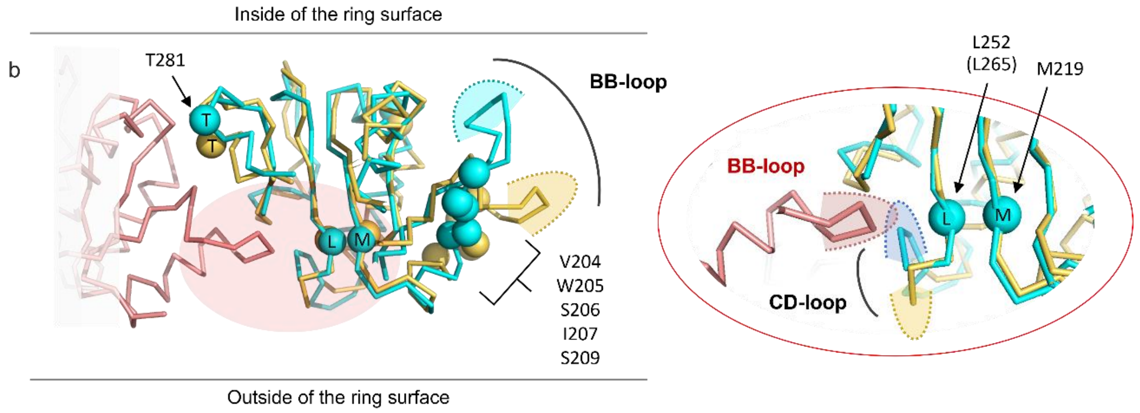

c

TIRMyD88 oligomer (cryo-EM structure)

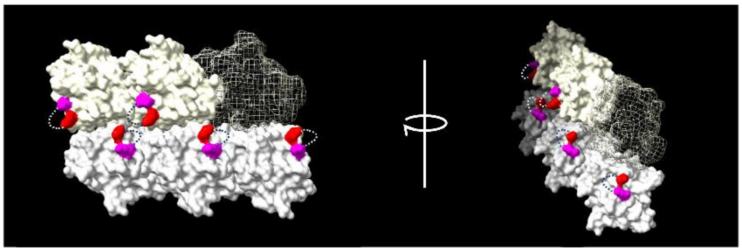

	Res	BSA/ASA
■ (red)	P245	0.00
⬚	G246	no density
⬚	A247	
⬚	H248	
■ (magenta)	Q249	0.00

MAL seeded TIRMyD88 oligomer (SFX structure)

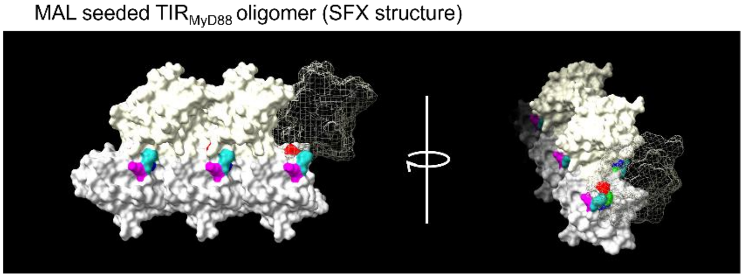

	Res	BSA/ASA
■ (red)	P245	0.96
■ (green)	G246	0.76
■ (blue)	A247	0.00
■ (cyan)	H248	0.16
■ (magenta)	Q249	0.00

Supplementary Fig. 5: Detailed intermolecular interactions and structural mapping of oncogenic mutations in TIR_{MyD88}. Related to Fig. 3

(a, b) Detailed comparison of the cryo-EM structure of oligomeric state of TIR_{MyD88} in the double-stranded filament with its monomeric state (see Supplementary Movie 2). Views from the outer surface of the double-layered ring (a) and the interfilament interface (b) are depicted. In (b), the red-ellipsed region is enlarged on the right. The areas enclosed by the dotted curves represent the tips of the BB loop and CD loop in monomeric or oligomeric state. During self-assembly, the BB loop undergoes reorganization, leading to both the intra- and interstrand interactions. Gain-of-function mutation residues (V204, W205, S206, I207, S209, M219, L252, and T281 in our study) associated with aggressive B-cell lymphoma are represented as spheres [7–9]. In the antiparallel double-stranded filament, residues within the BB loop (V204-I207 and S209) are situated at the interstrand interface, suggesting their role in head-to-head dimerization. Three other residues (M219, L252, and T281) are positioned at or close to the intrastrand interface. Thus, all of the oncogenic mutations mentioned above occur in positions crucial for maintaining the antiparallel double-stranded filament structure. This suggests that these oncogenic mutations strongly promote filament formation [10] by enhancing either inter- or intrastrand interactions, or both, resulting in unregulated downstream activation.

(c) Surface representations of TIR_{MyD88} oligomers highlighting differences in interstrand interface accessibility. Upper panel: TIR_{MyD88} oligomer in an antiparallel double-stranded filament (cryo-EM). Lower panel: Mal-induced TIR_{MyD88} oligomer in a parallel double-stranded filament (SFX; PDB 7BER). CD loop residues—P245 (red), G246 (lime), A247 (blue), H248 (light sea green), and Q249 (magenta)—are highlighted. Tables excerpted from Supplementary Table 3 summarize the involvement of each residue in the molecular interface; stronger interactions are indicated by darker red shading. BSA, buried surface area; ASA, accessible surface area; BSA/ASA, ratio of buried to accessible areas. One subunit of the TIR_{MyD88} oligomer is shown as a mesh representation. Notably, the accessible surface of the CD loop, particularly at P245, differs markedly; in the SFX structure, P245 is largely buried, whereas in the cryo-EM structure, it is fully solvent-exposed.

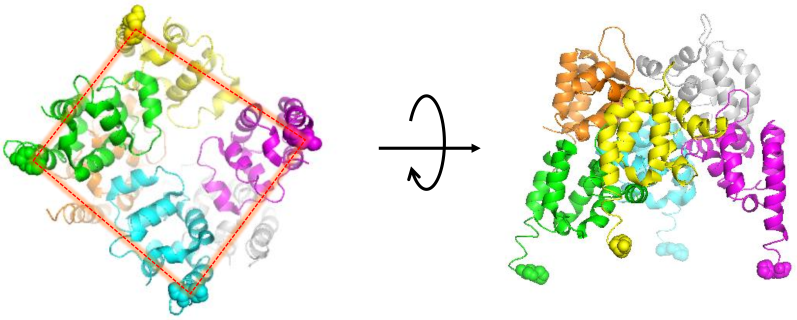

Supplementary Fig. 6: Four DD_{MyD88} subunits make a quasi-square in the helical oligomer. Related to Fig. 3 and Fig. 7

Six DD_{MyD88} subunits extracted from the helical complex with DD_{IRAK4} and DD_{IRAK2} are shown (PDB ID: 3MOP) [11]. The sphere models indicate the C-terminal residues of four DD_{MyD88} subunits in the bottom layer, to which TIR_{MyD88} are connected via linker residues (the intermediate domain). A quasi-square connecting the C-termini of DD_{MyD88} is represented by a red dotted line.

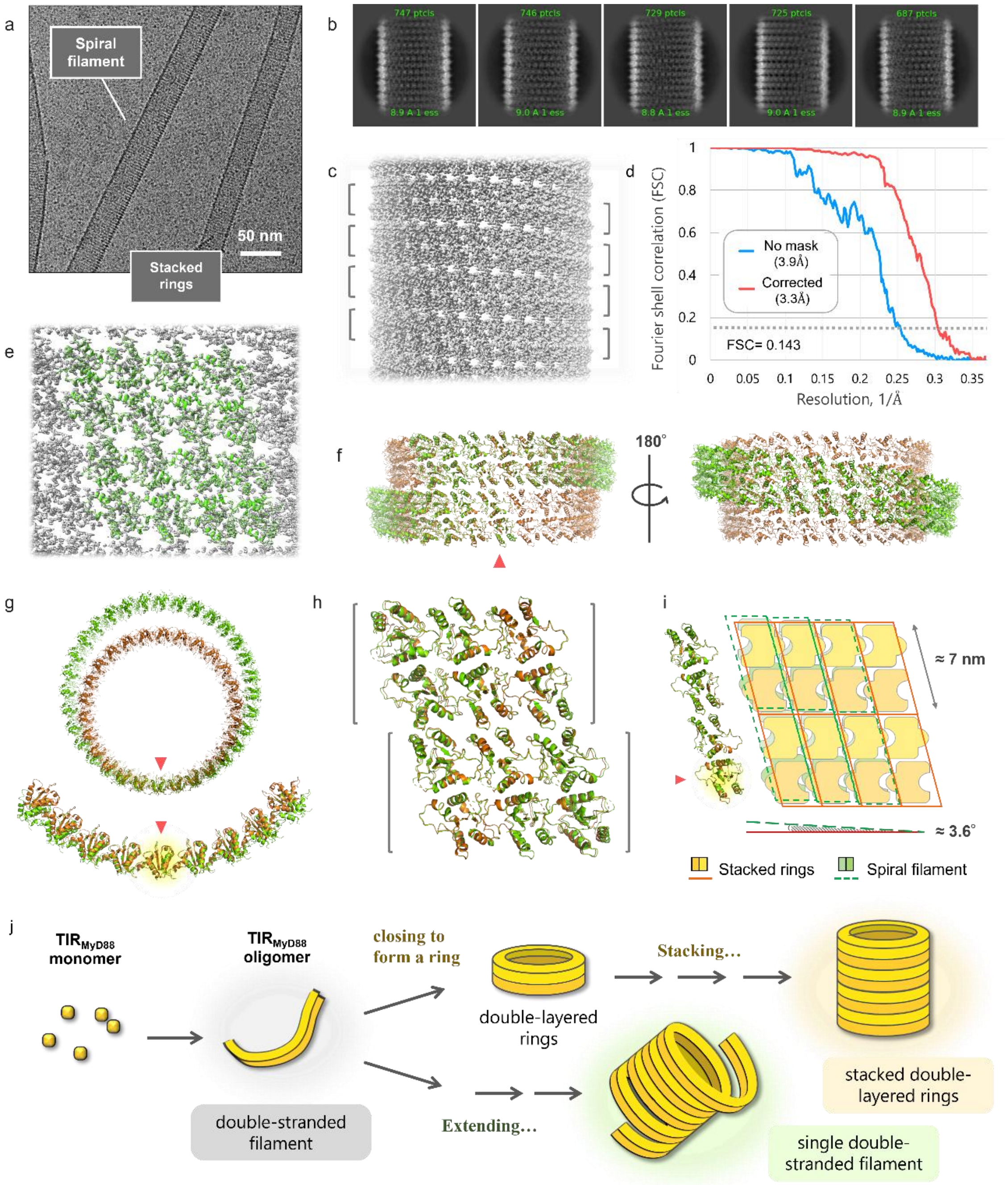

Supplementary Fig. 7: Spiral cylinder; overall and subunit arrangement. Related to Fig. 3

(a) An example of a cryo-EM micrograph showing two distinct cylindrical fibers of different diameters; stacked rings (see Fig. 3 and Supplementary Fig. 2) and spiral filament. (b) Representative 2D class averaged images of the spiral filament. (c) Cryo-EM density map of the cylindrical fiber formed by the spiral double-stranded filament. Gray brackets indicate the width of the double-stranded filament. (d) FSCs of (c) between two 3D structures from each half of the dataset, with and without masking. Based on the golden standard criterion, the resolution was 3.3 Å with a threshold of 0.143. (e) Molecular models fitted to the EM map. (f, g) A comparison of subunit arrangements in

the stacked-ring filament (orange) and the spiral filament (green) is depicted. Side (f) and top views (g) of the two cylindrical fibers are presented. The two cylinders are aligned using a subunit indicated by red triangles. (h) Superposition of 12 subunits in the stacked-ring and the spiral filament. Gray brackets indicate the width of the double-stranded filaments (i.e. double-layered ring). (i) Comparison of the subunit arrangements in the two filaments (side view). The subunit arrangement is essentially the same for both filaments. In particular, the vertical positioning of the subunits is nearly identical (left: four vertically arrayed subunits are aligned with the subunit indicated by the red triangle). However, their lateral positioning slightly differs, resulting in a tilt of approximately 3.6 degrees. This tilt creates the difference, either the stacked rings or the spiral filament. (j) Schematic of stacked rings and spiral cylinder formation. The spiral cylinder is formed from a single antiparallel double-stranded filament wound into a spiral. The two types of cylinders are formed from a common filament. The difference is whether the filament closes to form a ring or extends infinitely in a spiral.

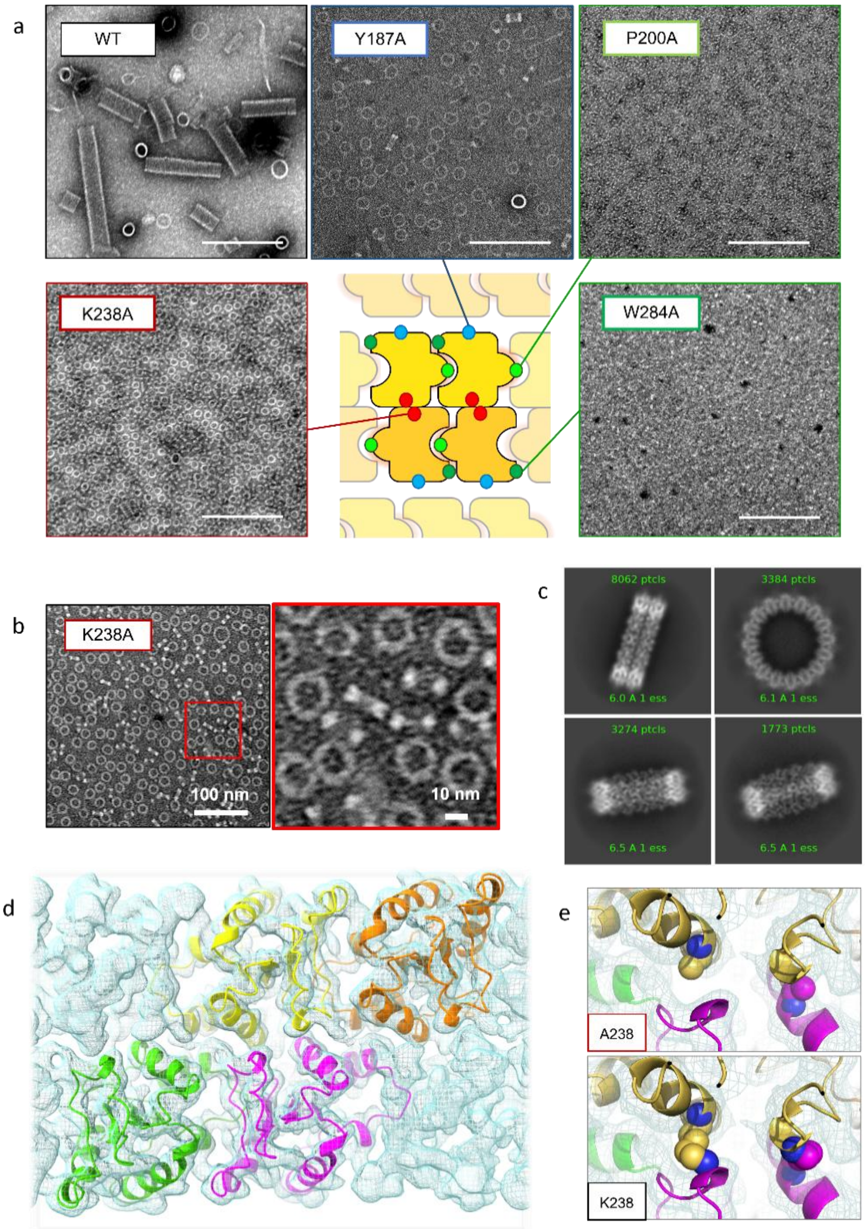

Supplementary Fig. 8: Self-assemble propensity of various TIR_{MyD88} mutants. Related to Fig. 4

(a) TEM images of WT TIR_{MyD88} or mutants of TIR_{MyD88}. The scale bar in (a) represents 200 nm. The positions of the mutated residues are shown in the cartoon. (b) TEM and (c) class-averaged cryo-EM images of the K238A rings. (d) Four molecular models of K238A TIR_{MyD88} fitted into the K238A EM map (FSC resolution was 3.2 Å) are shown. (e) A closer look at the sidechains (spheres) of two A238 residues from two subunits in (d) is shown (top). Replacing these Ala residues with Lys (as in the WT sequence) would result in collisions with the adjacent subunits (bottom). Representative images from at least three independent experiments with similar results are shown.

As shown in (a), mutants of TIR_{MyD88} with the P200A or W284A (intrastrand mutation) completely failed to self-assemble, while the Y187A mutant (interfilament mutation) formed rings similar to those formed by WT TIR_{MyD88}, but largely failed to form cylindrical fibers. The K238A mutant (interstrand mutation) formed rings but not cylindrical fibers. Notably, the diameter of the K238A mutant rings is significantly smaller, ranging from 14–25 nm (b), compared to the WT rings (22–38 nm). In fact, cryo-EM analysis of the K238A mutant rings revealed that the subunit arrangement is different from the WT TIR_{MyD88} ring (d). Importantly, such an arrangement is incompatible with WT TIR_{MyD88} due to steric clash of the K238 sidechains with adjacent subunits as shown in (e). This means that ectopically expressed K238A TIR_{MyD88} molecules in 293T cells as in Fig. 4b are essentially unable to co-assemble with endogenous WT MyD88, even if the K238A TIR_{MyD88} molecules can form homooligomers on their own. Therefore, dominant negative inhibition by the K238A mutant is not expected, which is consistent with the result shown in Fig. 4b.

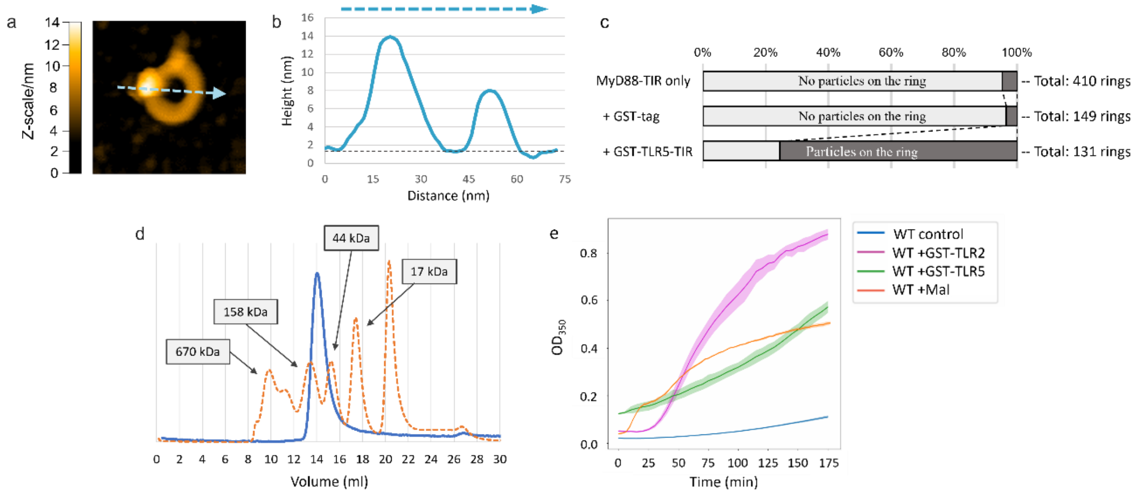

Supplementary Fig. 9: Direct visualization of the binding of GST-TIR$_{TLR5}$ to the TIR$_{MyD88}$ rings. Related to Fig. 6

(a) Averaged AFM image of WT rings bound by GST-TIR$_{TLR5}$. Scan area: 100 × 100 nm^2 with 100 × 100 pixels. Scan speed: 230 ms per image. (b) Sectional view along the dashed arrow in (a). (c) Frequency of topping events. The dark areas of the bars indicate the percentages of rings with particles among the total number of rings. Rings with particles were frequently observed when GST-TIR$_{TLR5}$ was added, whereas they were not observed when only either GST or TIR$_{TLR5}$ was added. (d) Analytical size-exclusion chromatography of GST-TIR$_{TLR2}$ (HiLoad® 10/300 Superdex® 75 pg: Cytiva). The blue solid and orange dotted lines indicate the elution profile of GST-TIR$_{TLR2}$ (~45 kDa) and molecular weight standards (Gel Filtration Standard: Bio-Rad), respectively. GST-TIR$_{TLR2}$ was eluted at ~91 kDa (14 mL), confirming homodimerization. (e) Turbidity assays of TIR$_{MyD88}$ (130 µM) upon 5% addition of GST-TIR$_{TLR2}$, GST-TIR$_{TLR5}$, or TIR $_{Mal}$. TIR$_{MyD88}$ was first dispensed into the plate, followed by the rapid addition of GST-TIR$_{TLR2}$, GST-TIR$_{TLR5}$, or TIR $_{Mal}$, and measured immediately. Data are presented as mean turbidity of duplicate experiments. Shaded bands indicate the range between minimum and maximum values. Representative image from at least three independent experiments with similar results are shown. Source data are provided as a Source Data file.

While TIR$_{TLR2}$ has been reported to form stable homodimers [12,13] and TLR2 has been proposed to function as a homodimer [14,15], TLR2 generally functions as heterodimers with TLR1 or TLR6 during signaling *in vivo*. Also, TLR1/2 and TLR2/6 employ the other adaptor protein, Mal, in addition to MyD88 [16]. To gain more direct insight into biological relevance, we examined TIR$_{TLR5}$ for MyD88 binding, because TLR5 functions exclusively as a homodimer and does not require Mal for signaling *in vivo* [16,17]. HS-AFM (a-c) showed that GST-TIR$_{TLR5}$ binds to preformed rings similarly to GST-TIR$_{TLR2}$.

The immediate turbidity increase upon GST-TIR_{TLR5} addition (OD_{350} at time zero) suggests that dimeric GST-TIR_{TLR5} triggers TIR_{MyD88} oligomerization more strongly than did dimeric GST-TIR_{TLR2}. This observation may explain why Mal is not required for TLR5 signaling; the GST-TIR_{TLR5}–TIR_{MyD88} interaction alone is sufficient to initiate oligomerization, whereas Mal-dependent TLR systems may require Mal to effectively facilitate this process.

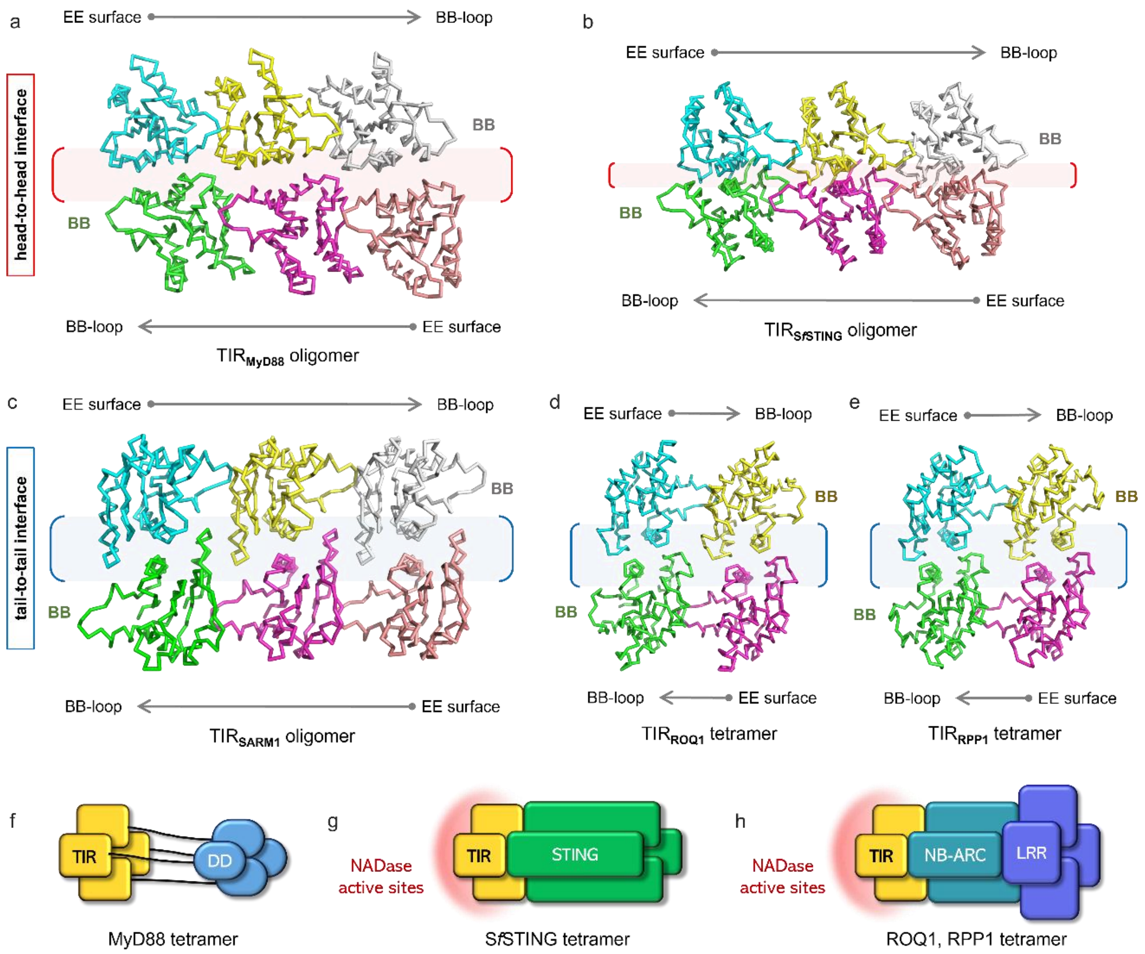

Supplementary Fig. 10: Comparison of the subunit arrangement in the double-stranded filament of TIR_{MyD88} with other TIR assemblies

(a, b) The subunit arrangement in the antiparallel double-stranded filament of TIR_{MyD88} determined in this work (a) and the *Sf* TIR-STING filament (b) from the antiphage effector protein of *Sphingobacterium faecium* are shown [18]. The homo-dimeric TIR-STING forms filaments upon binding to the nucleotide second messenger c-di-GMP at the STING domain. TIR_{STING} subsequently exhibits enzymatic activity to cleave NAD^+. In the filaments, homodimers of TIR_{STING} are arrayed laterally and the resulting subunit arrangement is topologically identical to that of TIR_{MyD88}. The lateral interactions between the subunits are mediated through the BB loop and the EE surface, just like the intrastrand interactions in the TIR_{MyD88} filaments. In both TIR_{MyD88} and TIR_{STING} filaments, the two strands are antiparallel, interacting through the interstrand interface. However, in the TIR_{STING} filament, the interstrand interactions are much more extensive, occupying ~16% (~1250 Å^2) of the total surface area of one subunit, compared to those in the TIR_{MyD88} filament, which occupy ~5% (~440 Å^2).

(c) The subunit arrangement in the TIR_{hSARM1} filament reveals that the two strands align antiparallel and associate through the interfilament interface [19].

(d, e) TIR_{ROQ1} and TIR_{RPP1} both form tetramers rather than extended filamentous assemblies [20,21]. Nevertheless, the arrangement of the tetrameric subunits is essentially the same as that of the TIR_{hSARM1} filament in (c).

(f-h) Schematic representation of tetramers of TIR-containing proteins, MyD88 (f), *Sf* TIR-STING (g), and ROQ1/RPP1 (h). It has been reported that the tetramerization of TIR_{STING}, TIR_{ROQ1}, and TIR_{RPP1} occurs cooperatively with the tetramerization of their co-existing effector domains (g, h). Hence, the cooperative character of tetramerization is likely conserved in these evolutionarily distant TIR-containing proteins. This strongly supports the idea that tetramerization of TIR_{MyD88} (f), triggered by activated TLRs/IL-1R, promotes self-assembly of DD_{MyD88}, which leads to the assembly of downstream IRAKs.

Supplementary Tables

Supplementary Table 1 | Cryo-EM data collection and structure refinement. Related to Fig. 3, Supplementary Fig. 3 and 7

	EMD-39676<br>PDB ID 8YYM	EMD-37355<br>PDB ID 8W8M
**Data Collection**		
Microscope	JEOL CRYO ARM 300	
Voltage (kV)	300	
Detector	GATAN K3 (6k x 4k)	
Recording mode	CDS	
Magnification	50,000	
Movie/micrograph pixel size (Å)	1.01	
Dose rate ($e^-/Å^2$)	1	
Defocus range (μm)	-0.5 to -2.0	
**EM Data Processing**		
Number of movies/micrographs	5,325	
Box size (pixel)	500 (1.010 Å/pixel)	450 (1.347 Å/pixel)
Initial particle number	60,578	253,118
Particle number (post 2D)	26,680	14,934
Particle number (for final map)	26,680	14,934
Symmetry	C26	C1
Helical rise (Å)	66.6	2.1
Helical twist (°)	-9.2	-11.4
Map resolution (FSC 0.143)	3.3	3.3
**Refinement and Validation**		
**Initial model Used**	7BER	7YRC
**Model Composition**		
Non-hydrogen protein atoms	115856	113934
Protein residues	14040	13770
Ligands	0	0
**RMSD from ideal**		
Bond length (Å)	0.006	0.002

Bond angles (°)	0.702	0.442
**Validation**		
Molprobity score	1.44	1.53
Clashscore	2.82	4.90
Rotamers outliers (%)	0.78	0.02
FSC (0.5) model-vs-map	3.3	3.46
CC model-vs-map (masked)	0.81	0.85
**Ramachandran Plot**		
Favored (%)	94.66	96.06
Allowed (%)	5.34	3.94
Outliers (%)	0	0

Supplementary Table 2 : Comparison of residues contributing to the intrastrand interfaces in the cryo-EM and SFX structures using the PISA program.

The relationships and extent of interactions among structures (subunits) A and B are shown. Stronger interactions are indicated by a deeper red shading. BSA, Buried Surface Area (Å²); ASA, Accessible Surface Area (Å²); B/A, BSA/ASA (ratio of buried to accessible area); HB, Hydrogen bonds.

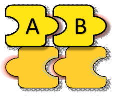

cryo-EM structure in this work							
		intrastrand interface					
		subunit A			subunit B		
residues		BSA	B/A	interaction	BSA	B/A	Interaction
C	168	0			0		
P	169	16.92	0.16	N278(HB)	0		
S	170	0			0		
D	171	0			0		
I	172	52.72	0.89		0		
Q	173	21.45	0.14		0		
F	174	0			0		
V	175	0			0		
Q	176	49.31	0.53	C280(HB)	0		
E	177	0			0		
M	178	0			0		
I	179	11.72	0.37		0		
R	180	0			0		
C	192	0			0		
V	193	5.33	0.82		0		
S	194	2.08	0.05		0		
D	195	63.02	0.60	R288(HB)	0		
R	196	55.9	0.46		0		
D	197	0			0		
V	198	28.17	0.61	R288(HB)	0		
L	199	96.15	0.68		0		
P	200	130.46	0.99		0		
G	201	75.13	0.89	T272(HB)	0		
T	202	46.87	0.62	T272(HB)	0		
C	203	8.95	0.12		0		
V	204	58.46	0.79	F270(HB)	0		
W	205	38.47	0.22		0		
S	206	0			0		
I	207	9.2	0.28		0		
A	208	0			0		
P	245	0			0		
G	246	no density					
A	247	no density					
H	248	no density					
Q	249	0			0		
K	250	0			81.64	0.57	
R	251	0			0		
L	252	0			4.66	0.30	
I	253	0			21.41	0.95	
P	254	0			0.67	0.57	
I	255	0			0		
L	268	0			0		
R	269	0			3.93	0.03	
F	270	0			75.92	0.43	V204(HB)
I	271	0			27.86	0.45	
T	272	0			85.99	0.68	G201(HB) T202(HB)
V	273	0			1.1	0.02	
C	274	0			11.42	1.00	
D	275	0			0		
T	276	0			0		
T	277	0			0		
N	278	0			30.54	0.46	P169(HB)
P	279	0			0		
C	280	0			73.94	0.66	Q176(HB)
T	281	0			28.44	0.72	
K	282	0			0		
S	283	0			0		
W	284	0			106.43	0.68	
F	285	0			0		
W	286	0			0		
T	287	0			0		
R	288	0			98.51	0.77	D195(HB) V198(HB)
L	289	0			2.5	0.71	
A	290	0			0		
K	291	0			18.54	0.14	
A	292	0			27.97	0.88	
L	293	0			0		
S	294	0			0		
L	295	0			18.07	0.22	
P	296	0			0		

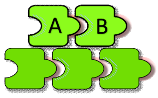

SFX structure (PDB ID: 7BER)							
		intrastrand interface					
		subunit A			subunit B		
residues		BSA	B/A	interaction	BSA	B/A	interaction
C	168	0			0		
P	169	12.5	0.11		0		
S	170	0			0		
D	171	0			0		
I	172	38.82	0.84		0		
Q	173	11.94	0.10		0		
F	174	0			0		
V	175	0			0		
Q	176	38.06	0.42		0		
E	177	0			0		
M	178	0			0		
I	179	7.7	0.43		0		
R	180	0			0		
C	192	0			0		
V	193	4.66	0.93		0		
S	194	5.33	0.14		0		
D	195	75.82	0.66		0		
R	196	64.7	0.56		0		
D	197	0			0		
V	198	25.08	0.79	R288(HB)	0		
L	199	109.35	0.85		0		
P	200	131.3	0.99		0		
G	201	89.27	1.00		0		
T	202	53.78	0.95	T272(HB)	0		
C	203	47.81	0.66		0		
V	204	38.46	0.56		0		
W	205	16.6	0.15		0		
S	206	22.12	0.37		0		
I	207	6.7	0.22		0		
A	208	0			0		
P	245	0			0		
G	246	0			12.31	0.25	
A	247	0			21.56	0.77	
H	248	0			19.26	0.11	
Q	249	0			0		
K	250	0			72.14	0.53	
R	251	0			0.44	0.01	
L	252	0			5.32	0.44	
I	253	0			18.4	0.98	
P	254	0			0.17	1.00	
I	255	0			0		
L	268	0			0		
R	269	0			3.44	0.02	
F	270	0			59.13	0.46	
I	271	0			16.11	0.74	
T	272	0			87.42	0.76	T202(HB)
V	273	0			1.09	0.03	
C	274	0			9.45	1.00	
D	275	0			0		
T	276	0			0		
T	277	0			0		
N	278	0			15.7	0.27	
P	279	0			0		
C	280	0			56.19	0.51	
T	281	0			40.2	0.85	
K	282	0			0		
S	283	0			0		
W	284	0			108.68	0.69	
F	285	0			0		
W	286	0			0		
T	287	0			0		
R	288	0			103.33	0.87	V198(HB)
L	289	0			1.18	1.00	
A	290	0			0		
K	291	0			18.06	0.14	
A	292	0			28.01	0.85	
L	293	0			0		
S	294	0			0		
L	295	0			9.37	0.13	
P	296	0			0		

Supplementary Table 3 : Comparison of residues contributing to the interstrand interfaces in the cryo-EM and SFX structures using the PISA program.

The relationships and extent of interactions among structures (subunits) A (or B) and C are shown. Stronger interactions are indicated by a deeper red shading. BSA, Buried Surface Area (Å²); ASA, Accessible Surface Area (Å²); B/A, BSA/ASA (ratio of buried to accessible area); HB, Hydrogen bonds.

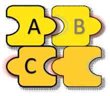

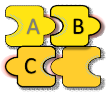

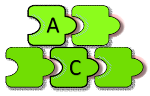

cryo-EM structure in this work													
		interstrand interface						diagonal interaction interface					
		subunit A			subunit C			subunit B			subunit C		
residues		BSA	B/A	interaction	BSA	B/A	Interaction	BSA	B/A	interaction	BSA	B/A	Interaction
T	202	0			0			0			0		
C	203	0.82	0.01		0			0			0		
V	204	0			0			0			0		
W	205	106.64	0.61	S242(HB)	101.24	0.58		0			0		
S	206	0			0			0			0		
I	207	0			0			0			0		
A	208	3.51	0.68		3.25	0.71		0			0		
S	209	15.33	0.24		18.19	0.28		0			0		
E	210	0			0			0			0		
L	211	0			0			0			0		
I	212	0			0			0			0		
E	213	0			0			0			0		
K	214	0			0			0			0		
S	230	0			0			0			0		
K	231	14.74	0.09		12.31	0.07		0			0		
E	232	0			0			0			0		
C	233	0			0			0			0		
D	234	0			0			0			0		
F	235	72.16	0.86		72.28	0.85		0			0		
Q	236	0			0			0			0		
T	237	0			0			0			0		
K	238	69.87	0.59		70.06	0.60		0			0		
F	239	39.86	0.71		42.33	0.75		0			0		
A	240	0			0			0			0		
L	241	29.61	0.41		32.06	0.44		0.17	0.00		0.33	0.00	
S	242	71.83	0.82		75.1	0.86	W205(HB)	0			0		
L	243	0			0			0			0		
S	244	2.58	0.03		6.5	0.07		0			0		
P	245	0			0			0			0		
G	246	no density						no density					
A	247	no density						no density					
H	248	no density						no density					
Q	249	0			0			0			0		
P	265	0			0			0			0		
S	266	0			0			26.38	0.37	R269(HB)	24.72	0.34	R269(HB)
I	267	10.45	0.11		9.36	0.10		38.42	0.39		37.19	0.38	
L	268	0			0			0			0		
R	269	0			0			44.79	0.32	S266(HB)	45.04	0.32	S266(HB)
F	270	0			0			44.59	0.25		47.03	0.27	
I	271	0			0			0			0		

SFX structure (PDB ID: 7BER)							
		interstrand interface					
		subunit A			subunit C		
residues		BSA	B/A	interaction	BSA	B/A	interaction
T	202	0			0		
C	203	0			24.26	0.34	
V	204	0			0		
W	205	0			93.42	0.85	
S	206	0			19.25	0.32	
I	207	0			0		
A	208	0			0		
S	209	0			20.11	0.68	
E	210	0			0		
L	211	0			0		
I	212	0			0		
E	213	0			28.74	0.34	R269(HB)
K	214	0			0		
S	230	0			0		
K	231	0			43.58	0.27	
E	232	0			1.01	0.01	
C	233	0			0		
D	234	0			30	0.53	H248(HB)
F	235	0			69.72	1.00	
Q	236	0			0		
T	237	0			0		
K	238	47.15	0.45		57.99	0.55	
F	239	0			44.29	0.99	
A	240	0			0		
L	241	26.86	0.96		0		
S	242	19.47	0.27		40.64	0.57	
L	243	9.51	0.19		16.67	0.34	
S	244	1.34	0.11		0		
P	245	87.83	0.96		0		
G	246	36.06	0.75		0		
A	247	0			0		
H	248	27.34	0.16	D234(HB)	0		
Q	249	0			0		
P	265	0			0		
S	266	34.67	0.52		0		
I	267	57.09	0.78		0		
L	268	0			0		
R	269	84.19	0.47	E213(HB)	0		
F	270	69.83	0.54		0		
I	271	0			0		

Supplementary Table 4 : Comparison of residues contributing to the interfilament interfaces in the cryo-EM using the PISA program.

The relationships and extent of interactions among structures (subunits) A and D are shown. Stronger interactions are indicated by a deeper red shading. BSA, Buried Surface Area (Å²); ASA, Accessible Surface Area (Å²); B/A, BSA/ASA (ratio of buried to accessible area); HB, Hydrogen bonds.

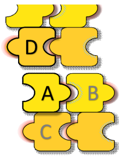

cryo-EM structure in this work							
		interfilament interface					
		subunit A			subunit D		
residues		BSA	B/A	interaction	BSA	B/A	Interaction
I	179	0			0		
R	180	28.23	0.23		24.36	0.20	
Q	181	51.59	0.68	N186(HB)	0		
L	182	0.67	0.24		0		
E	183	0			0		
Q	184	2.58	0.02		67.97	0.51	
T	185	43.98	0.65		50.6	0.74	
N	186	7.36	0.06		110.08	0.94	Q181(HB)
Y	187	42.78	0.37	R188(HB)	27.07	0.23	
R	188	0			56.57	0.31	Y187(HB)
L	189	0			0		
N	278	0			0		
P	279	2.81	0.03		0		
C	280	0			0		
T	281	0			0		
K	282	33.71	0.36		0		
S	283	45.17	0.51		3.31	0.04	
W	284	0			0		
F	285	0			0		
W	286	43.45	0.71		0		
T	287	10.32	0.14		20.71	0.28	
R	288	0			0		
L	289	0			0		
A	290	1.18	0.51		0		
K	291	0			0		

Supplementary Reference

1. Punjani, A., Rubinstein, J. L., Fleet, D. J. & Brubaker, M. A. cryoSPARC: algorithms for rapid unsupervised cryo-EM structure determination. *Nat. Methods* **14**, 290–296 (2017).
2. Kucukelbir, A., Sigworth, F. J. & Tagare, H. D. Quantifying the local resolution of cryo-EM density maps. *Nat. Methods* **11**, 63–65 (2014).
3. Clabbers, M. T. B. *et al.* MyD88 TIR domain higher-order assembly interactions revealed by microcrystal electron diffraction and serial femtosecond crystallography. *Nat. Commun.* **12**, 2578 (2021).
4. Ve, T. *et al.* Structural basis of TIR-domain-assembly formation in MAL- and MyD88-dependent TLR4 signaling. *Nat. Struct. Mol. Biol.* **24**, 743–751 (2017).
5. Ohnishi, H. *et al.* Structural basis for the multiple interactions of the MyD88 TIR domain in TLR4 signaling. *Proc. Natl. Acad. Sci. USA* **106**, 10260–10265 (2009).
6. Snyder, G. A. *et al.* Molecular mechanisms for the subversion of MyD88 signaling by TcpC from virulent uropathogenic Escherichia coli. *Proc. Natl. Acad. Sci. USA* **110**, 6985–6990 (2013).
7. Ngo, V. N. *et al.* Oncogenically active MYD88 mutations in human lymphoma. *Nature* **470**, 115–119 (2011).
8. Yu, X. *et al.* MYD88 L265P Mutation in Lymphoid Malignancies. *Cancer Res.* **78**, 2457–2462 (2018).
9. de Groen, R. A. L., Schrader, A. M. R., Kersten, M. J., Pals, S. T. & Vermaat, J. S. P. MYD88 in the driver's seat of B-cell lymphomagenesis: from molecular mechanisms to clinical implications. *Haematologica* **104**, 2337–2348 (2019).

10. O'Carroll, A. *et al.* Pathological mutations differentially affect the self-assembly and polymerisation of the innate immune system signalling adaptor molecule MyD88. *BMC Biol.* **16**, 149 (2018).

11. Lin, S.-C., Lo, Y.-C. & Wu, H. Helical assembly in the MyD88–IRAK4–IRAK2 complex in TLR/IL-1R signalling. *Nature* **465**, 885–890 (2010).

12. Xu, Y. *et al.* Structural basis for signal transduction by the Toll/interleukin-1 receptor domains. *Nature* **408**, 111–115 (2000).

13. Tao, X., Xu, Y., Zheng, Y., Beg, A. A. & Tong, L. An extensively associated dimer in the structure of the C713S mutant of the TIR domain of human TLR2. *Biochem. Biophys. Res. Commun.* **299**, 216–221 (2002).

14. Zheng, H. *et al.* The TLR2 is activated by sporozoites and suppresses intrahepatic rodent malaria parasite development. *Sci. Rep.* **5**, 18239 (2015).

15. Bagheri, V., Askari, A., Arababadi, M. K. & Kennedy, D. Can Toll-Like Receptor (TLR) 2 be considered as a new target for immunotherapy against hepatitis B infection? *Hum. Immunol.* **75**, 549–554 (2014).

16. Horng, T., Barton, G. M., Flavell, R. A. & Medzhitov, R. The adaptor molecule TIRAP provides signalling specificity for Toll-like receptors. *Nature* **420**, 329–333 (2002).

17. Voogdt, C. G. P., Wagenaar, J. A. & van Putten, J. P. M. Duplicated TLR5 of zebrafish functions as a heterodimeric receptor. *Proc. Natl. Acad. Sci. USA* **115**, E3221–E3229 (2018).

18. Morehouse, B. R. *et al.* Cryo-EM structure of an active bacterial TIR-STING filament complex. *Nature* **608**, 803–807 (2022).

19. Shi, Y. *et al.* Structural basis of SARM1 activation, substrate recognition, and inhibition by small molecules. *Mol. Cell* **82**, 1643–1659.e10 (2022).

20. Martin, R. *et al.* Structure of the activated ROQ1 resistosome directly recognizing the pathogen effector XopQ. *Science* **370**, eabd9993 (2020).

21. Ma, S. *et al.* Direct pathogen-induced assembly of an NLR immune receptor complex to form a holoenzyme. *Science* **370**, eabe3069 (2020).
