# Supplementary material for: Structural Mechanism of Receptor-Triggered MyD88 Oligomeric Assembly in Innate Immune Signaling
Source: Nat Commun. 2026 Apr 17;17:5364. doi: 10.1038/s41467-026-71836-8 (PMC13276377; doi:10.1038/s41467-026-71836-8)
Supplement: Supplementary file 2 — Description of Additional Supplementary Files [file 41467_2026_71836_MOESM2_ESM.pdf]

Description of Additional Supplementary Files

File name: Supplementary Movie 1
Description: HS-AFM movie of disintegration and regeneration of TIR_{MyD88} rings. Related to Fig. 5.
Scan area: 60 × 60 nm^2 with 120 × 120 pixels. Scan speed: 600 ms per image.

File name: Supplementary Movie 2
Description: Structural comparison of TIR_{MyD88} in monomeric and oligomeric states. Related to Fig. 7d, Supplementary Fig. 5a and 5b. The spheres represent Cα atoms of amino acid residues that are frequently mutated in aggressive B-cell lymphoma.

File name: Supplementary Movie 3
Description: HS-AFM movie of GST-TIR_{TLR2} binding to TIR_{MyD88} rings. Related to Fig. 6.
Scan area: 200 × 200 nm^2 with 120 × 120 pixels. Scan speed: 500 ms per image.
