# Supplementary material for: Structural Mechanism of Receptor-Triggered MyD88 Oligomeric Assembly in Innate Immune Signaling
Source: Nat Commun. 2026 Apr 17;17:5364. doi: 10.1038/s41467-026-71836-8 (PMC13276377; doi:10.1038/s41467-026-71836-8)
Supplement: Supplementary file 6 — Reporting Summary [file 41467_2026_71836_MOESM6_ESM.pdf]

nature portfolio

Corresponding author(s): Hidehito Tochio

Last updated by author(s): Feb 26, 2026

Reporting Summary

Nature Portfolio wishes to improve the reproducibility of the work that we publish. This form provides structure for consistency and transparency in reporting. For further information on Nature Portfolio policies, see our Editorial Policies and the Editorial Policy Checklist.

Statistics

For all statistical analyses, confirm that the following items are present in the figure legend, table legend, main text, or Methods section.

n/a	Confirmed	
☐	☒	The exact sample size (*n*) for each experimental group/condition, given as a discrete number and unit of measurement
☐	☒	A statement on whether measurements were taken from distinct samples or whether the same sample was measured repeatedly
☐	☒	The statistical test(s) used AND whether they are one- or two-sided<br>*Only common tests should be described solely by name; describe more complex techniques in the Methods section.*
☒	☐	A description of all covariates tested
☐	☒	A description of any assumptions or corrections, such as tests of normality and adjustment for multiple comparisons
☐	☒	A full description of the statistical parameters including central tendency (e.g. means) or other basic estimates (e.g. regression coefficient) AND variation (e.g. standard deviation) or associated estimates of uncertainty (e.g. confidence intervals)
☐	☒	For null hypothesis testing, the test statistic (e.g. *F*, *t*, *r*) with confidence intervals, effect sizes, degrees of freedom and *P* value noted<br>*Give P values as exact values whenever suitable.*
☒	☐	For Bayesian analysis, information on the choice of priors and Markov chain Monte Carlo settings
☒	☐	For hierarchical and complex designs, identification of the appropriate level for tests and full reporting of outcomes
☒	☐	Estimates of effect sizes (e.g. Cohen's *d*, Pearson's *r*), indicating how they were calculated

Our web collection on statistics for biologists contains articles on many of the points above.

Software and code

Policy information about availability of computer code

Data collection	Cryo-EM: SerialEM and YoneoLocr for data collection.<br>HS-AFM: a laboratory-built software developed based on IgorPro ver.6.
Data analysis	Cryo-EM: CryoSPARC (v3.2.0), Chimera (v1.17.1), ChimeraX (v1.7.1) , Coot (0.9.4.1), ResMap (x1.1.4).<br>HS-AFM: Kodec v4.7.5.0.<br>Data plot: Python, Microsoft Excel.

For manuscripts utilizing custom algorithms or software that are central to the research but not yet described in published literature, software must be made available to editors and reviewers. We strongly encourage code deposition in a community repository (e.g. GitHub). See the Nature Portfolio guidelines for submitting code & software for further information.

Data

Policy information about availability of data

All manuscripts must include a data availability statement. This statement should provide the following information, where applicable:

- Accession codes, unique identifiers, or web links for publicly available datasets
- A description of any restrictions on data availability
- For clinical datasets or third party data, please ensure that the statement adheres to our policy

The coordinates have been deposited in the Protein Data Bank (PDB) under accession code: MyD88-TIR oligomer forming stacked rings–8YYM and helical cylinder–

8W8M, and the EM maps have been deposited in the Electron Microscopy Data Bank (EMDB) (EMD-39676 and EMD-37355). All data relevant to the conclusions presented within the manuscript are included, with additional data available upon further request.

Research involving human participants, their data, or biological material

Policy information about studies with human participants or human data. See also policy information about sex, gender (identity/presentation), and sexual orientation and race, ethnicity and racism.

Reporting on sex and gender	n/a
Reporting on race, ethnicity, or other socially relevant groupings	n/a
Population characteristics	n/a
Recruitment	n/a
Ethics oversight	n/a

Note that full information on the approval of the study protocol must also be provided in the manuscript.

Field-specific reporting

Please select the one below that is the best fit for your research. If you are not sure, read the appropriate sections before making your selection.

☒ Life sciences ☐ Behavioural & social sciences ☐ Ecological, evolutionary & environmental sciences

For a reference copy of the document with all sections, see nature.com/documents/nr-reporting-summary-flat.pdf

Life sciences study design

All studies must disclose on these points even when the disclosure is negative.

Sample size	Cryo-EM data: Sample sizes were not predetermined for this study. 2D classes were used as a guide to determine the sample sizes for the map calculation.<br>HS-AFM: Sample sizes were determined on the basis of previous experience to evaluate the reproducibility. No statistical methods were used to predetermine sample size.<br>Turbidity assays were performed in triplicate (Fig.1 and Fig.6) or in duplicate (Supplementary Fig.9e).<br>Dominant negative assay (Fig.4) was performed in triplicate.
Data exclusions	HS-AFM: molecules that are obviously aggregated or broken under the observation were excluded.
Replication	Cryo-EM data were collected from each dataset and processed as described.<br><br>HS-AFM images were reprensentative of repeated experiments.<br><br>The tubidity assays were poerformed in triplicate or duplicate. No experiments were excluded.<br><br>The dominant negative assays were performed in triplicate. No experiments were excluded.
Randomization	No randomization was attempted or needed.
Blinding	Investigators were not blinded. No blinding was needed for this study.

Reporting for specific materials, systems and methods

We require information from authors about some types of materials, experimental systems and methods used in many studies. Here, indicate whether each material, system or method listed is relevant to your study. If you are not sure if a list item applies to your research, read the appropriate section before selecting a response.

Materials & experimental systems

n/a	Involved in the study
☒	☐ Antibodies
☐	☒ Eukaryotic cell lines
☒	☐ Palaeontology and archaeology
☒	☐ Animals and other organisms
☒	☐ Clinical data
☒	☐ Dual use research of concern
☒	☐ Plants

Methods

n/a	Involved in the study
☒	☐ ChIP-seq
☒	☐ Flow cytometry
☒	☐ MRI-based neuroimaging

Eukaryotic cell lines

Policy information about cell lines and Sex and Gender in Research

Cell line source(s)	HEK293T (RIKEN BRC)
Authentication	The HEK293T cell line was purchased from RIKEN BRC. The cell line was not further authenticated in our laboratory.
Mycoplasma contamination	All cells were tested and shown to be mycoplasma-free.
Commonly misidentified lines (See ICLAC register)	No cell lines used appear on the register of commonly misidentified cell lines.

Plants

Seed stocks	*Report on the source of all seed stocks or other plant material used. If applicable, state the seed stock centre and catalogue number. If plant specimens were collected from the field, describe the collection location, date and sampling procedures.*
Novel plant genotypes	*Describe the methods by which all novel plant genotypes were produced. This includes those generated by transgenic approaches, gene editing, chemical/radiation-based mutagenesis and hybridization. For transgenic lines, describe the transformation method, the number of independent lines analyzed and the generation upon which experiments were performed. For gene-edited lines, describe the editor used, the endogenous sequence targeted for editing, the targeting guide RNA sequence (if applicable) and how the editor was applied.*
Authentication	*Describe any authentication procedures for each seed stock used or novel genotype generated. Describe any experiments used to assess the effect of a mutation and, where applicable, how potential secondary effects (e.g. second site T-DNA insertions, mosiacism, off-target gene editing) were examined.*
