# Supplementary material for: Structural Mechanism of Receptor-Triggered MyD88 Oligomeric Assembly in Innate Immune Signaling
Source: Nat Commun. 2026 Apr 17;17:5364. doi: 10.1038/s41467-026-71836-8 (PMC13276377; doi:10.1038/s41467-026-71836-8)
Supplement: Supplementary file 7 — Transparent Peer Review file [file 41467_2026_71836_MOESM7_ESM.pdf]

nature portfolio

Peer Review File

Structural mechanism of receptor-triggered MyD88 oligomeric assembly in innate immune signaling

Corresponding Author: Professor Hidehito Tochio

This file contains all reviewer reports in order by version, followed by all author rebuttals in order by version.

Version 0:

Reviewer comments:

Reviewer #1

(Remarks to the Author)
Review: From Monomers to Oligomers: Structural Mechanism of Receptor-Triggered MyD88 Assembly in Innate Immune Signaling

Summary: In the study, the authors solved the structure of oligomers of the purified MyD88-TIR domain using cryo-electron microscopy. The purified TIR domain could assemble into filaments and higher-order rings that could stack into cylindrical fibers. Using high-speed AFM, the authors could directly observe the addition of the MyD88-TIR domain to these filaments, thereby directly observing oligomerization. The authors also observed that the TLR2-TIR domains are preferably bound to one filament interface, suggesting a structural basis for how TLRs trigger MyD88 oligomerization. I believe the core advance of the paper is that the MyD88-TIR domain can oligomerize in the absence of the Mal/TIRAP TIR domain (unfortunately, this isn't clearly stated). This sets the work apart from other structural studies of MyD88 (see reference 1,2). However, this point needs further experimental work to strengthen this claim (see below).

In general, I find the experiments to be well-executed and convincing. What is missing is a concise and clear statement on this paper's conceptual advance. Second, the authors should clearly spell out that the cylindrical rings are most likely not a physiologically relevant structure but an in vitro artifact. I know this statement is briefly mentioned in the discussion, but a clearer statement should be made. I would strongly recommend streamlining the discussion, it is currently too long.

I would also suggest the following experiments:

1. Repeating the experiments in Figure 6 with a receptor TIR that does not require Mal/TIRAP would strengthen the core conceptual advance. TLR2 is believed to require TIRAP/Mal for signaling(ref 3). I would recommend the IL-1R receptor. Here, the authors must make a heterodimer of IL-1R-TIR and IL-1AcP-TIR domains. I believe TLR5 does not require Mal and could be an alternative.

2. A comparison of the MyD88 TIR oligomer in the presence of Mal-TIR would be informative and increase the impact of the work. One question this work raises is: what is the precise role of TIRAP? Does it potentiate MyD88 assembly? If the authors could provide some structural details on this other TIR domain protein and how it changes MyD88 oligomerization kinetic or structure, it would be helpful to the community.

1. Clabbers, M. T. B. et al. MyD88 TIR domain higher-order assembly interactions revealed by microcrystal electron diffraction and serial femtosecond crystallography. Nat. Commun. 12, 2578 (2021).
2. Ve, T. et al. Structural basis of TIR-domain-assembly formation in MAL- and MyD88-dependent TLR4 signaling. Nat. Struct. Mol. Biol. 24, 743–751 (2017).
3. Yamamoto, M. et al. Essential role for TIRAP in activation of the signalling cascade shared by TLR2 and TLR4. Nature 420, 324–329 (2002).

Reviewer #2

(Remarks to the Author)

The major finding of this study includes an observed in vitro head to tail antiparallel MyD88 TIR domain arrangement which forms double-stranded cylindrical filaments and ring structures. Additionally, AFM used to for monitoring MyD88 TIR reassembly is novel.

As noted by the authors, several previous NMR, X-ray, MicroED crystal and cryoEM structures of MyD88 TIR domains observed as individual in solution monomers, as crystal lattice monomers, oligomers and filamented assemblies have been previously reported. The MyD88 TIR filaments reported here, also as noted by the authors, are similar to previously reported, with exception of the head to tail antiparallel configuration. Which is an interesting observation. Although, it is not immediately clear what the physiological relevance of the antiparallel head to tail arrangement is over previously observed MAL/TIRAP seeded TIR-MyD88 assembly.

Given that the authors make the effort to use GST-TIR homodimers of TIR2 and TIR5 to charcterize how receptor mediated GST TIR dimers affect MyD88 TIR assembly and reassembly using AFM, it would be interesting to see in solution how receptor or adaptor TIR dimers affect MyD88-TIR assembly in solution. One potential experiment that could potentially address observed differences between could be to use existing GST- receptor TIR dimers (GST-TIR2 or GST-TIR5) in comparison to MAL either alone or as GST-MAL dimer to see how this affects MyD88 TIR head to tail or unidirectional assembly. It is not clear other than the construct and the concentration of MyD88-TIR what is mediating this head to tail antiparallel assembly in absence of either receptor or adaptor TIR domain.

An attempt is made to functionally verify the observed antiparallel double-stranded filament arrangement using NF-κB cell signaling. The use of AFM showing dynamic assembly and reassembly of MyD88-TIR is novel and could be potentially useful as tool for mechanistic studies.

Reviewer #3

(Remarks to the Author)
The author used biochemical and structure biological approach to study the mechanism involved in the oligomerization of MyD88 mediated by its TIRMyD88 domain. The author found that TIRMyD88 is able to self-assembles into filaments and can further form ring and cylindrical fibers. Such self-assembles was independent on Mal, while addition of GST-tagged receptor TIRs accelerated the self-assembly. Author suggested that addition of receptor TIRs mimics the signal initiation in vivo. Besides, CryoEM analysis further revealed the structure details of ring and cylindrical as a result of self-assembly. They also applied High-speed atomic force microscopy to visualize the dynamic processes of oligomerization of TIRMyD88. According to these results, they proposed a regulatory mechanism of TIRMyD88 oligomerization. This is an interesting work and provides some novel insights, however, major issues must be addressed prior acceptance to ensure the conclusion is well supported by experimental data.
Major questions:
1. It is interesting to see the differences between self-oligomerization vs Mal-induced oligomerization. Do the authors think both structures are biologically relevant? If so, how the structural differences fit in the functional differences of MYD88 in Mal dependent and independent pathways? The authors should discuss this.
2. The author should include the model/map FSC for thinner filament and thicker filament.
3. Page 14, Author examined the mutations at intra-strand, inter-strand and inter-filament residues and proposed that antiparallel double-stranded filament structure represents the functional oligomeric state of TIRMyD88 in cells. How do those results support self-assembled antiparallel oligomers over parallel Mal-induced oligomers? It might be helpful to map the mutations here in the previous structure of Mal-induced oligomer to see if they might also affect the mal-induced oligomerization. Besides, are there disease mutations that only affect one mechanism but not the other?
4. Page 5, line 123, line 210, line 823: The author discussed gain of function residues that are associated with B-cell lymphoma in expanded data, including V204, W205, S206, I207, S209, M219, L252, and T281. Is there data that shows how mutation at those residues affect the overall oligomer assembly, especially L252P. Considering these mutations are so important regarding clinical application, it would be good to include those data and discuss along with cancer progression.
5. Page 12, line 323: In order to draw the conclusion that "TIRMyD88 is thought to co-assemble with the endogenous MyD88" More data are required to validate the co-assembly, such as pull-down assay followed by SDS-PAGE/Western Blot to visualize both endogenous and ectopical variant of MyD88.
6. Page 17 Line 440, line 485: “Because GST exists as a dimer, it is assumed that TIRTLR2 is forced to dimerize in the corresponding fusion proteins. Thus, these results indicate that only dimeric TIRTLR2 binds to TIRMyD88 rings.” It is true that GST tends to form dimers, but it does not mean the entire fusion protein will also be dimerized. More data are needed to verify the dimerization of purified GST- TIRTLR2, such as SEC along with standard proteins.
7. Please provided side-chain density in Fig 4a.
Minor points:
8. Page 8, line 198: why use short incubation (5 -10 mins) on the AFM stage, is this due to the mechanical forces? Will there be cylindrical fibers formed with a longer incubation time?
9. Page 9, line 210: why the residue number are different? (L265P, L252 in this study) Any deletion in the construct used in this study?
10. Page 12, line 328: “A similar effect may occur here.” Need to validate it experimentally.
11. Page 14 Line 346: “Negative stain TEM revealed that TIRMyD88 with the W284A mutation (located on the EE surface) completely failed to self-assemble (Extended Data Fig 6a). Thus, the reduced dominant-negative effects are likely attributable to the reduced self-oligomerization ability of TIRMyD88.” The reduced dominant-negative effects were caused by the reduced self-assembly or co-assembly with the endogenous MyD88, or both?
12. Page 17 Line 435, “First, 434 TIRMyD88 rings were prepared on the AFM stage (Fig. 6a), and GST-TIRTLR2 was

added” please add the concentration of the GST-TIRTLR2.
13. Page 18, Line 479: “In addition, the amount of GST-TIRTLR2 required for the promotion of self-assembly was only 5% that of TIRMyD88, strongly suggesting that GST-TIRTLR2 is required only at the initial stage of assembly.” How did the author determine the amount of TIRMyD88 that works (5%)? Does lower amount also induce the self-assembly, and what is the limit?
14. Page 46, line 1032: “For experimental samples of GST-TIRTLR2/TLR5, the expression vectors were transformed into Escherichia coli BL-21 (DE3) (Novagen).” Should be “The cells were transformed with the expression vectors.”

Reviewer #4

(Remarks to the Author)
I co-reviewed this manuscript with one of the reviewers who provided the listed reports. This is part of the Nature Communications initiative to facilitate training in peer review and to provide appropriate recognition for Early Career Researchers who co-review manuscripts.

Reviewer #5

(Remarks to the Author)
This manuscript presents a highly insightful investigation into the self-assembly of MyD88 and its interactions with Toll-like receptors (TLRs), using an impressive combination of cryo-electron microscopy (cryo-EM) and high-speed atomic force microscopy (HS-AFM). The study provides valuable structural and dynamic insights into the oligomerization of MyD88, which is crucial for understanding its role in signal initiation and immune response regulation.

The manuscript is well written, with a clear flow of ideas and a logical presentation of the experiments. The experimental design is robust and the results are convincing. The use of both cryo-EM and HS-AFM to observe the MyD88 oligomerization process is particularly commendable, as it offers complementary perspectives on the static and dynamic aspects of these molecular complexes. The findings significantly advance our understanding of MyD88’s role in immune signaling and its potential implications in various pathologies, including B-cell lymphomas and autoinflammatory diseases.

With a background in biophysics, my reviews focus more on the AFM experiments, the HS-AFM experiments are carefully executed, and the correlation between the HS-AFM and cryo-EM results strongly supports the authors' conclusions. The dynamic visualization of oligomerization using HS-AFM is particularly exciting, as it sheds light on the regulatory mechanisms governing this process.

One minor point for clarification: the authors mention (lines 199-200) that "in AFM, mechanical forces are applied to the specimen; thus, only molecular assemblies that are somewhat resistant to deformation are visualized." It would be beneficial if the authors could provide an estimation of the forces applied during imaging and discuss how these forces might influence the measured height values. Are the height values corrected for potential deformation, and how does this impact the interpretation of the structural data?

Another minor comment is related to Figure 4b: In the caption, the authors should display the individual data points and clearly explain what is represented (e.g., means ± standard deviation). This will help ensure transparency in the data presentation.

Version 1:

Reviewer comments:

Reviewer #1

(Remarks to the Author)
The authors have revised their manuscript with additional experimental data and clearer text. The work provides important insights into the structural properties of the TIR domain. The authors propose that this structure facilitates the nucleation and assembly of death domain (DD) complexes. In particular, they clearly explain why they believe the MyD88-TIR structures explain how TIR receptors that do not require MAL trigger MyD88 oligomerization and Myddosome assembly.

We believe the major strength lies in a structural rationale linking TIR interfaces to DD assembly, particularly in the absence of MAL. The added data and improved clarity strengthen the central claims. The inclusion of GST–TIR–TLR5 experiments provides a valuable comparison to TLR2 (a receptor that signals with MAL).

Major comments: The cellular validation of TIR-domain mutants remains the weakest part. To strengthen physiological relevance, we would like to see the authors test the mutants in the context of full-length MyD88 reconstituted into a MyD88-null line (e.g., immortalized MyD88–/– macrophages from BEI Resources), with appropriate expression controls. This system could then be used to compare signaling responses downstream of MAL-dependent TLRs (e.g., TLR2, TLR4) with those downstream of MAL-independent TLRs (e.g., TLR5), to contextualize the importance of the residues identified at the MyD88-TIR fiber interface.

Recommendation: Minor revisions. While additional cellular experiments would further strengthen the manuscript, they are not essential for publication and could be addressed in subsequent work by this or other groups.

Reviewer #3

(Remarks to the Author)
All previous concerns were resolved. Required data were added to the supplementary. The current conclusion is well supported by the data presented in the manuscript.

Reviewer #4

(Remarks to the Author)
I co-reviewed this manuscript with one of the reviewers who provided the listed reports. This is part of the Nature Communications initiative to facilitate training in peer review and to provide appropriate recognition for Early Career Researchers who co-review manuscripts.

Reviewer #5

(Remarks to the Author)
The authors have responded to my comments in a satisfactory manner.
Congrats for the nice work
David Alsteens

Open Access This Peer Review File is licensed under a Creative Commons Attribution 4.0 International License, which permits use, sharing, adaptation, distribution and reproduction in any medium or format, as long as you give appropriate credit to the original author(s) and the source, provide a link to the Creative Commons license, and indicate if changes were made.
In cases where reviewers are anonymous, credit should be given to 'Anonymous Referee' and the source.
The images or other third party material in this Peer Review File are included in the article's Creative Commons license, unless indicated otherwise in a credit line to the material. If material is not included in the article's Creative Commons license and your intended use is not permitted by statutory regulation or exceeds the permitted use, you will need to obtain permission directly from the copyright holder.
To view a copy of this license, visit https://creativecommons.org/licenses/by/4.0/

Responses to Reviewers

Response to Reviewer #1

We would like to thank the reviewer for the careful assessment of our study and for their constructive comments, which have greatly improved our manuscript.

Summary: In the study, the authors solved the structure of oligomers of the purified MyD88-TIR domain using cryo-electron microscopy. The purified TIR domain could assemble into filaments and higher-order rings that could stack into cylindrical fibers. Using high-speed AFM, the authors could directly observe the addition of the MyD88-TIR domain to these filaments, thereby directly observing oligomerization. The authors also observed that the TLR2-TIR domains are preferably bound to one filament interface, suggesting a structural basis for how TLRs trigger MyD88 oligomerization. I believe the core advance of the paper is that the MyD88-TIR domain can oligomerize in the absence of the Mal/TIRAP TIR domain (unfortunately, this isn't clearly stated). This sets the work apart from other structural studies of MyD88 (see reference 1,2). However, this point needs further experimental work to strengthen this claim (see below).

In general, I find the experiments to be well-executed and convincing. What is missing is a concise and clear statement on this paper's conceptual advance. Second, the authors should clearly spell out that the cylindrical rings are most likely not a physiologically relevant structure but an in vitro artifact. I know this statement is briefly mentioned in the discussion, but a clearer statement should be made. I would strongly recommend streamlining the discussion, it is currently too long.

Response-1: We thank the reviewer for the positive assessment of our experiments and the constructive suggestions.

To clarify the conceptual advance of the paper, we have added a statement to the Introduction (page 6, line 140) explicitly noting that our study provides novel molecular insights into the assembly mechanisms of MyD88 in the absence of Mal. This is critical for understanding Mal-independent TLR and IL-1R signaling pathways.

"This study elucidates the mechanistic basis of a previously uncharacterized Mal-independent route to MyD88 activation, wherein receptor dimerization directly nucleates TIRMyD88 filaments that are structurally distinct from Mal-induced assemblies."

Regarding the cylindrical structures, we state in the Discussion (page 22, line 598) that these morphologies are in vitro artifacts, as follows.

*"Because the cylindrical fibers of TIRMyD88 take much longer to form than do the rings (Fig. 1a) and the interfilament mutants do not significantly affect the dominant negative inhibitory effect (Fig. 4b), **these cylindrical assemblies may represent an in vitro artifact.**"*

We have substantially streamlined the Discussion section as recommended, reducing its length to improve clarity and focus, particularly by markedly shortening the section on *"An evolutionarily conserved self-assembly mode of the TIR domain"*.

I would also suggest the following experiments:
1. Repeating the experiments in Figure 6 with a receptor TIR that does not require Mal/TIRAP would strengthen the core conceptual advance. TLR2 is believed to require TIRAP/Mal for signaling(ref 3). I would recommend the IL-1R receptor. Here, the authors must make a heterodimer of IL-1R-TIR and IL-1AcP-TIR domains. I believe TLR5 does not require Mal and could be an alternative.

Response-2: Thank you for the suggestion. As suggested by the reviewer, we conducted a turbidity assay for MyD88-TIR using GST-TLR5-TIR (Extended Data Fig. 7e). The results clearly showed that the addition of a small amount of GST-TLR5-TIR (5% of MyD88-TIR) boosted an increase in the turbidity of the MyD88-TIR solution.

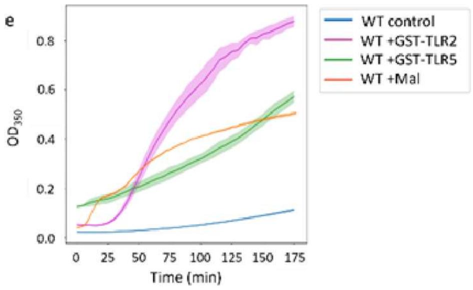

As a distinctive feature of GST-TLR5-TIR, the turbidity increased immediately upon the addition of MyD88-TIR; note the elevated OD_{350} at time zero. This observation suggests that dimeric TLR5-TIR has a stronger ability to initiate MyD88-TIR oligomerization than does dimeric TLR2-TIR. This could be one reason for the dispensability of Mal in TLR5 signaling. Thus, the affinity of dimeric TLR5 for MyD88 was sufficient to trigger MyD88 oligomerization. In contrast, in Mal-dependent TLR signaling, the receptors alone are insufficient to quickly trigger MyD88 oligomerization.

Note that isolated TLR5-TIR was not examined in this study. This is because a sufficient amount of isolated TLR5-TIR could not be obtained because of its aggregation-prone nature after removal of the GST-tag. Nevertheless, the new data clearly demonstrated that dimeric TLR5-TIR triggered MyD88-TIR oligomerization without Mal.

2. A comparison of the MyD88 TIR oligomer in the presence of Mal-TIR would be informative and increase the impact of the work. One question this work raises is: what is the precise role of TIRAP? Does it potentiate MyD88 assembly? If the authors could provide some structural details on this other TIR domain protein and how it changes MyD88 oligomerization kinetic or structure, it would be helpful to the community.

1. Clabbers, M. T. B. et al. MyD88 TIR domain higher-order assembly interactions revealed by microcrystal electron diffraction and serial femtosecond crystallography. Nat. Commun. 12, 2578 (2021).
2. Ve, T. et al. Structural basis of TIR-domain-assembly formation in MAL- and MyD88-dependent TLR4 signaling. Nat. Struct. Mol. Biol. 24, 743–751 (2017).
3. Yamamoto, M. et al. Essential role for TIRAP in activation of the signalling cascade shared by TLR2 and TLR4. Nature 420, 324–329 (2002).

Response-3: As indicated by the reviewer, Mal-TIR can "*potentiate MyD88 assembly*." We conducted a turbidity assay of MyD88-TIR, in which monomeric Mal-TIR was added (Extended Data Fig. 7e, also shown in **Response-2** above). The results demonstrated that addition of a small amount of Mal-TIR (5% of MyD88-TIR) substantially enhanced the oligomerization of MyD88-TIR. Similar results were reported previously (ref. Ve et al. *Nature Struct Mol Biol* (2017)). Notably, upon the addition of Mal-TIR, MyD88-TIR immediately started oligomerization, whereas there was a time lag

when dimeric TLR2-TIR was added. Thus, in Mal-dependent TLR signaling, such as that mediated by TLR2, the receptors alone may be insufficient to promptly trigger MyD88 oligomerization.

The key structural difference between the two oligomers lies in the parallel versus antiparallel mode of the strand assembly. The parallel arrangement of Mal-induced oligomers was primarily determined by Mal-TIR assembly. During the crystallization of MyD88-TIR in the presence of Mal-oligomers (acting as templates), the MyD88-TIR subunits were preferentially aligned according to this template structure because of the structural similarity between the Mal-TIR and MyD88-TIR domains. In contrast, the antiparallel mode is considered to be the inherent assembly mode of MyD88-TIR.

Response to Reviewer #2

We would like to thank the reviewer for the careful assessment of our study and for their constructive comments, which have greatly improved our manuscript.

The major finding of this study includes an observed in vitro head to tail antiparallel MyD88 TIR domain arrangement which forms double-stranded cylindrical filaments and ring structures. Additionally, AFM used to for monitoring MyD88 TIR reassembly is novel.

As noted by the authors, several previous NMR, X-ray, MicroED crystal and cryoEM structures of MyD88 TIR domains observed as individual in solution monomers, as crystal lattice monomers, oligomers and filamented assemblies have been previously reported. The MyD88 TIR filaments reported here, also as noted by the authors, are similar to previously reported, with exception of the head to tail antiparallel configuration. Which is an interesting observation. Although, it is not immediately clear what the physiological relevance of the antiparallel head to tail arrangement is over previously observed MAL/TIRAP seeded TIR-MyD88 assembly.

Response-1: Thank you for the comment. Before addressing the specific points, I would like to clarify a minor misunderstanding in the review. To date, no cryo-EM structure of MyD88-TIR, in the monomeric or oligomeric form, has been reported. This study presents the first cryo-EM structure of MyD88-TIR.

In several MyD88-dependent pathways, including those mediated by TLR5, IL-1, and IL-18, Mal is dispensable. A previously reported Mal-induced oligomer of MyD88-TIR was generated on Mal fibers that served as a template, producing a parallel assembly. Consequently, it remained unclear whether MyD88-TIR adopts the same parallel architecture in the absence of Mal oligomers. Our study demonstrated for the first time that MyD88 intrinsically self-assembles in an antiparallel configuration.

As described in Figure 7 and Extended Data Fig. 3, an important biochemical insight from the antiparallel arrangement of MyD88-TIR is its geometrical consistency in positioning the four DDs that can promote tetramerization. From this structure, it can be inferred that MyD88-TIR oligomerization drives MyD88-DD tetramerization in the full-length context.

In contrast, the parallel arrangement induced by Mal oligomers lacks the spatial requirements for optimal MyD88-DD tetramer formation. Thus, although the parallel mode can promote clustering of MyD88-DD, optimal positioning of MyD88-DD is not achieved. Consequently, the parallel mode is expected to drive Myddosome formation less efficiently than the antiparallel mode. The low efficiency of the parallel mode, however, might be compensated for by the strong clustering capacity of Mal in cells, which could facilitate the recruitment and oligomerization of additional MyD88 molecules. This interpretation remains speculative and requires further experimental validation.

We have added sentences describing this consideration on page 12, line 294, and page 20, line 530.

"In contrast, the parallel arrangement induced by Mal does not impose the spatial constraints required for optimal DD_{MyD88} tetramer formation. Thus, although the parallel mode can cluster DD_{MyD88}, it does not facilitate their optimal positioning for tetramerization."

"In contrast, the Mal-dependent parallel arrangement of TIR_{MyD88} oligomers lacks the spatial constraints that arrange DD_{MyD88} in optimal positions. This arrangement is expected to drive Myddosome formation less efficiently than by the antiparallel mode (see Results). Nevertheless, the strong clustering capacity of Mal in cells might offset this reduced efficiency, although this remains speculative and requires further experimental testing."

Given that the authors make the effort to use GST-TIR homodimers of TIR2 and TIR5 to charcterize how receptor mediated GST TIR dimers affect MyD88 TIR assembly and reassembly using AFM, it would be interesting to see in solution how receptor or adaptor TIR dimers affect MyD88-TIR assembly in solution. One potential experiment that could potentially address observed differences between could be to use existing GST- receptor TIR dimers (GST-TIR2 or GST-TIR5) in comparison to MAL either alone or as GST-MAL dimer to see how this affects MyD88 TIR head to tail or unidirectional assembly.

Response-2: We agree with the reviewer that the AFM analysis of MyD88-TIR assemblies formed in the presence of receptor TIRs or Mal is significant. However, the addition of Mal or GST-TLR2/5 to MyD88-TIR generated amorphous aggregates in addition to fibers, precluding the preparation of specimens suitable for AFM or cryo-EM. Consequently, we have not yet been able to conduct a rigorous analysis; this remains an important direction for future research.

It is not clear other than the construct and the concentration of MyD88-TIR what is mediating this head to tail antiparallel assembly in absence of either receptor or adaptor TIR domain.

Response-3: As stated in Response-1, MyD88 self-assembles intrinsically in an anti-parallel configuration in the absence of either the receptor or Mal.

As shown in Figure 1c, a lag phase precedes the increase in turbidity, indicating that nucleation is required before oligomerization can occur. Moreover, the lag phase was concentration-dependent, implying that oligomerization did not proceed effectively at low concentrations, where nucleation events were rare. Under physiological conditions, where MyD88 concentrations are even lower, nucleation events are even more infrequent, making spontaneous oligomerization unlikely. Therefore, we propose that dimeric receptors or Mal may accelerate this nucleation step. Consistent with this model, the addition of as little as 5% GST-TLR2/5-TIR or Mal-TIR markedly shortened the lag phase of MyD88 oligomerization (Fig. 6f and Extended Data Fig. 7e).

Notably, our turbidity assays used monomeric rather than oligomeric Mal-TIR. Therefore, MyD88-TIR oligomers generated under these conditions may differ from the parallel assemblies templated by Mal fibers. Because these samples have not yet been subjected to structural analysis, no definitive conclusions can be drawn. In summary, MyD88-TIR is intrinsically capable of nucleating antiparallel assemblies; however, this process is suppressed in cells because of the low intracellular abundance of MyD88. Engagement with dimeric receptor TIRs relieves this suppression and initiates nucleation-driven antiparallel oligomerization. However, the structural basis by which receptor-mediated interactions trigger nucleation remains to be elucidated.

An attempt is made to functionally verify the observed antiparallel double-stranded filament arrangement using NF-κB cell signaling. The use of AFM showing dynamic assembly and reassembly of MyD88-TIR is novel and could be potentially useful as tool for mechanistic studies.

Response-4: We thank the reviewer for acknowledging our functional verification via NF-κB signaling and for recognizing the novelty and potential utility of our AFM-based approach in elucidating the mechanistic assembly of MyD88-TIR.

Response to Reviewer #3

We would like to thank the reviewer for the careful assessment of our study and for their constructive comments, which have greatly improved our manuscript.

The author used biochemical and structure biological approach to study the mechanism involved in the oligomerization of MyD88 mediated by its TIRMyD88 domain. The author found that TIRMyD88 is able to self-assembles into filaments and can further form ring and cylindrical fibers. Such self-assembles was independent on Mal, while addition of GST-tagged receptor TIRs accelerated the self-assembly. Author suggested that addition of receptor TIRs mimics the signal initiation in vivo. Besides, CryoEM analysis further revealed the structure details of ring and cylindrical as a result of self-assembly. They also applied High-speed atomic force microscopy to visualize the dynamic processes of oligomerization of TIRMyD88. According to these results, they proposed a regulatory mechanism of TIRMyD88 oligomerization. This is an interesting work and provides some novel insights, however, major issues must be addressed prior acceptance to ensure the conclusion is well supported by experimental data.

Major questions:
1. It is interesting to see the differences between self-oligomerization vs Mal-induced oligomerization. Do the authors think both structures are biologically relevant? If so, how the structural differences fit in the functional differences of MYD88 in Mal dependent and independent pathways? The authors should discuss this.

Response-1: We thank the reviewer for the positive assessment of our experiments and the constructive suggestions.

In Mal-dependent pathways, Mal-TIR promotes MyD88-TIR oligomerization by serving as a template for assembly. In contrast, in Mal-independent pathways, such as those mediated by TLR5 or IL-1R, there is no need for MyD88-TIR to oligomerize in the same manner as Mal-TIR oligomers. Therefore, it is reasonable to assume that both structural configurations are biologically relevant to their respective pathways.

From a structural perspective, as stated in **Response-3** to reviewer #1, the antiparallel configuration (self-assembly mode) appears to be more efficient in promoting Myddosome formation owing to its geometrical advantage in positioning MyD88-DDs in addition to the clustering effect, whereas the parallel configuration (Mal-induced assembly mode) lacks this geometrical advantage and relies primarily on the clustering (and templating) effect. However, this disadvantage of the parallel configuration may be compensated for by the strong clustering capacity of Mal; as a result, the efficiency of Myddosome formation may not be significantly reduced. Additional experimental data are required to establish the biological significance of each configuration.

The following sentence, which discusses this point, has been added to the main text on page 20, line 530.

"In contrast, the Mal-dependent parallel arrangement of TIR_{MyD88} oligomers lacks the spatial constraints that arrange DD_{MyD88} in optimal positions. This arrangement is expected to drive Myddosome formation less efficiently than by the antiparallel mode (see Results). Nevertheless, the strong clustering capacity of Mal in cells might offset this reduced efficiency, although this remains speculative and requires further experimental testing."

2. The author should include the model/map FSC for thinner filament and thicker filament.

Response-2: The model/map FSC curves for these filaments have been included in Extended Data Fig. 2b and 5d in the revised manuscript.

3. Page 14, Author examined the mutations at intra-strand, inter-strand and inter-filament residues and proposed that antiparallel double-stranded filament structure represents the functional oligomeric state of TIRMyD88 in cells. How do those results support self-assembled antiparallel oligomers over parallel Mal-induced oligomers? It might be helpful to map the mutations here in the previous structure of Mal-induced oligomer to see if they might also affect the mal-induced oligomerization. Besides, are there disease mutations that only affect one mechanism but not the other?

Response-3: In the revised manuscript, we have included a table (Supplemental Table S2-4) showing the results of protein interface analysis using the protein interfaces, surfaces, and assemblies (PISA) program (Krissinel, E., and Henrick, K., *J. Mol. Biol.,* 372, 774(2007)). As shown in the table, the residues at the subunit interface are largely common between the parallel and antiparallel configurations, making it difficult to distinguish between the two configurations based on mutations in these residues. However, a remarkable difference is observed in the CD loop. In the antiparallel configuration (cryo-EM structure), this loop is exposed to the solvent and does not appear in the cryo-EM map. Therefore, it was not expected to contribute significantly to MyD88-TIR oligomer formation or concomitant signaling. In contrast, in the parallel Mal-induced oligomers (SFX structure), the CD loop was located at the subunit interface and was largely buried. This suggests that mutations in the CD loop affect oligomer formation and, consequently, signaling capacity. Different degrees of CD loop exposure are shown in Extended Data Fig. 3l in the revised manuscript (also shown below).

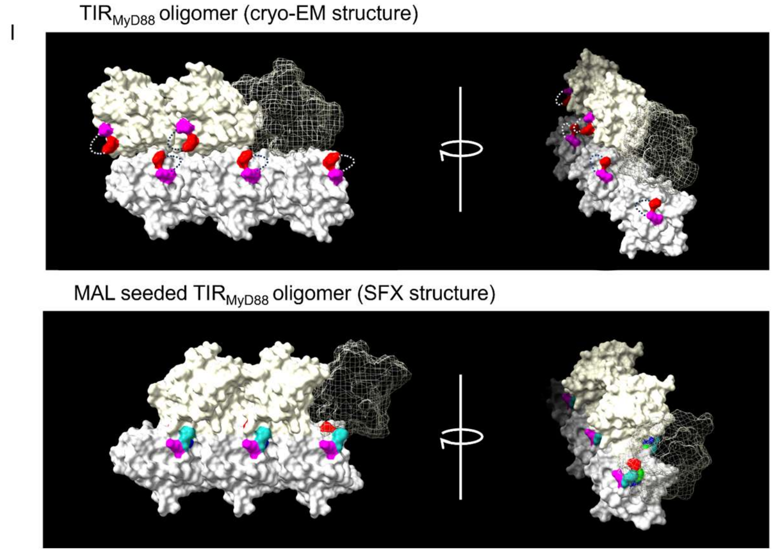

	Res	BSA/ASA
■ (red)	P245	0.00
⬚	G246	no density
⬚	A247	
⬚	H248	
■ (magenta)	Q249	0.00

	Res	BSA/ASA
■ (red)	P245	0.96
■ (green)	G246	0.76
■ (blue)	A247	0.00
■ (cyan)	H248	0.16
■ (magenta)	Q249	0.00

Our dominant negative assay, focusing on the CD loop (P245, G246, and H248), demonstrated that mutating the loop did not significantly affect NF-κB activation (Fig. 4b). This result strongly supports

the antiparallel configuration over the parallel configuration in the cells, which is consistent with the prediction that the CD loop is not involved in subunit interfaces in the antiparallel mode. Thus, our assay results favored the antiparallel mode over the parallel mode under physiological conditions. The assay was conducted using the IL-18 signaling pathway, which is independent of Mal.

The following sentence discussing this point has been added to the main text on page 15, line 379:

"Mutations at P245, G246 or H248 in the CD loop did not affect NF-κB activity, suggesting that the contribution of these residues to signal transduction is negligible (Fig. 4b). Consistently, these residues were exposed to the solvent in the oligomers, with little or no density map. In contrast, in the Mal-induced oligomers, these residues were buried at the interstrand interface (Supplementary Table2,3 and Extended Data Fig. 3l)"

"Besides, are there disease mutations that only affect one mechanism but not the other?"

We appreciate the reviewer's insightful question, which is indeed important for understanding the disease mechanisms. However, this aspect is still under investigation, and we do not yet have sufficient data to draw conclusions. We plan to conduct a systematic analysis of disease-associated variants in future studies.

4. Page 5, line 123, line 210, line 823: The author discussed gain of function residues that are associated with B-cell lymphoma in expanded data, including V204, W205, S206, I207, S209, M219, L252, and T281. Is there data that shows how mutation at those residues affect the overall oligomer assembly, especially L252P. Considering these mutations are so important regarding clinical application, it would be good to include those data and discuss along with cancer progression.

Response-4: Unfortunately, we do not currently have data demonstrating the effects of mutations at these specific residues on oligomer assembly. Given its clinical importance, particularly the L252P mutation, we prepared a protein carrying this mutation. However, we have encountered significant challenges owing to its strong propensity to aggregate, which has thus far prevented the successful purification and detailed analysis of oligomeric states. We will continue our research to overcome these purification challenges and clarify their effect on oligomer assembly and their clinical relevance.

5. Page 12, line 323: In order to draw the conclusion that "TIRMyD88 is thought to co-assemble with the endogenous MyD88" More data are required to validate the co-assembly, such as pull-down assay followed by SDS-PAGE/Western Blot to visualize both endogenous and ectopical variant of MyD88.

Response-5: We thank the reviewer for this suggestion. Although pull-down followed by SDS-PAGE/western blotting would be informative, our ectopic construct was the isolated MyD88 TIR domain. A previous study showed that isolated TIR domain does not pull down full-length MyD88 under standard co-IP conditions, indicating that TIR–TIR contacts are sensitive to dilution and detergents, with DD-mediated nucleation dominating these workflows (Pustelny et al., *Cell Commun. Signal.,* 20, 10 (2022)). Accordingly, co-IP with TIR-only constructs is prone to underestimating the interactions.

Our dominant-negative assay, a well-established readout of MyD88 function (e.g., Burns et al., *J. Biol. Chem.,* 273, 12203 (1998); Wesche et al., *Immunity,* 7, 837 (1997)), demonstrated that ectopic TIR_{MYD88} suppresses MyD88-dependent signaling in cells, which is consistent with competition at the multivalent interfaces required for productive TIR assembly. Together with our structural

observations of stable TIR oligomers under defined solution conditions, these data are consistent with the TIR-mediated higher-order assembly in the MyD88 pathway, even when direct visualization of the co-assembly is not feasible.

To avoid overinterpretation and align our wording with the evidence available in this study, we have toned down the statement on page 13, line 338:

"Thus, the ectopically expressed TIR_{MyD88}, which lacks DD_{MyD88}, likely competes for the multivalent TIR-TIR interfaces required for the productive assembly of endogenous MyD88, thereby preventing the formation of functional oligomers competent to recruit IRAK4."

6. Page 17 Line 440, line 485: "Because GST exists as a dimer, it is assumed that TIRTLR2 is forced to dimerize in the corresponding fusion proteins. Thus, these results indicate that only dimeric TIRTLR2 binds to TIRMyD88 rings." It is true that GST tends to form dimers, but it does not mean the entire fusion protein will also be dimerized. More data are needed to verify the dimerization of purified GST- TIRTLR2, such as SEC along with standard proteins.

Response-6: We addressed the reviewer's concern by including the SEC profile of GST-TLR2-TIR in the revised manuscript (Extended Data Fig. 7d). This SEC profile clearly demonstrated that the GST-fusion protein predominantly existed as a dimer in solution.

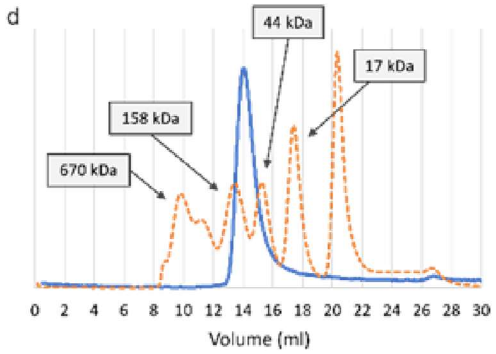

7. Please provided side-chain density in Fig 4a.

Response-7: To better illustrate the interactions at the interface, side-chain density has been included in Extended Data Fig. 2. Unfortunately, the confined space in Fig. 4a does not allow for its insertion.

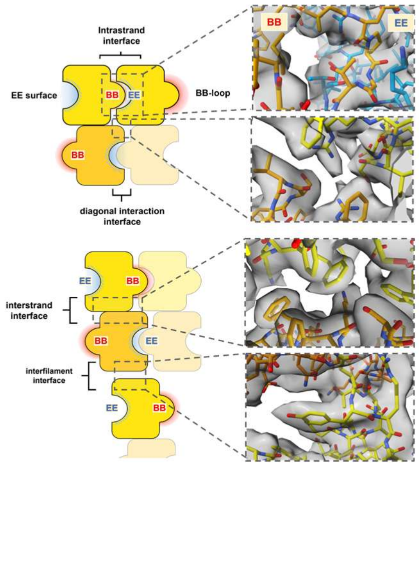

Minor points:

8. Page 8, line 198: why use short incubation (5 -10 mins) on the AFM stage, is this due to the mechanical forces? Will there be cylindrical fibers formed with a longer incubation time?

Response-8: We thank the reviewer for the insightful questions regarding the incubation time at the AFM stage and the potential formation of larger assemblies, such as cylindrical fibers.

We performed the experiments using longer incubation times during the AFM stage. However, we did not observe cylindrical fibers, although in rare cases, some sheet structures that could potentially form cylindrical shapes when closed were observed.

As discussed in **Response-1 to reviewer #5**, cylindrical fibers may be stable in solution yet insufficiently rigid to withstand the force exerted between the sample and the mica substrate during AFM imaging. We inferred that the interfilament interactions promoting cylinder formation were not sufficiently strong to resist this substrate force. The ring structures observed were consistently single- or double-layered, and rings with more than two layers were not detected, supporting this interpretation.

Therefore, an incubation time of 5- to 10-minutes was chosen as optimal for observing the predominant and relatively stable assembly species (primarily rings) that could be reliably deposited and imaged under our AFM experimental conditions.

9. Page 9, line 210: why the residue number are different? (L265P, L252 in this study) Any deletion in the construct used in this study?

Response-9: The difference in residue numbering (L265P vs. L252P) reflects an updated human MYD88 reference sequence. In the earlier RefSeq entry (NM_002468.4/NP_002459.2), leucine was numbered 265. In the current MANE/RefSeq sequence, NM_002468.5/NP_002459.3, the same leucine is numbered 252, whereas the amino acid sequence length (296 residues) was unchanged.

Because "L265P" remains widely used in the B-cell lymphoma literature, we refer to the variant as L252P (historically L265P) throughout the manuscript to maintain continuity with previous studies. No deletions or insertions were present in the construct used in this study.

To avoid confusion, the Methods section now states that all residue numbering follows RefSeq NP_002459.3 (MANE Select).

"The coding region of human TIR_{MyD88} (amino acids 153–296; numbering according to RefSeq NP_002459.3) was cloned into the pET-28a vector (Novagen), which was engineered with a 6×His-tagged streptococcal protein GB1 domain (GB1) for protein expression and purification. For the luciferase activity assay, TIR_{MyD88} (residues 148–296; numbering per NP_002459.3) with an N-terminus myc epitope was also cloned into the pcDNA3.1+ vector (Invitrogen)."

10. Page 12, line 328: "A similar effect may occur here." Need to validate it experimentally.

Response-10: We thank the reviewer for this comment. Consistent with our approach in Response-5, we avoided overinterpretation and revised the sentence to acknowledge that this mechanism was not directly tested, rather than adding new experiments. The revised sentence (page 13, line 343) is as follows:

" A similar effect may underlie our observations; however, we did not directly test this mechanism, and its operation remains to be demonstrated."

11. *Page 14 Line 346: "Negative stain TEM revealed that TIRMyD88 with the W284A mutation (located on the EE surface) completely failed to self-assemble (Extended Data Fig 6a). Thus, the reduced dominant-negative effects are likely attributable to the reduced self-oligomerization ability of TIRMyD88." The reduced dominant-negative effects were caused by the reduced self-assembly or co-assembly with the endogenous MyD88, or both?*

Response-11: We thank the reviewer for this insightful question.

As noted by the reviewer, *"attributable to the reduced self-oligomerization"* was inaccurate. Interactions with endogenous MyD88 are required to prevent signal transduction. Because the W284A mutation impairs the intrastrand TIR-TIR interaction (as shown by the abolished self-assembly in TEM), it reduces the ability of this mutant to co-assemble with endogenous MyD88 TIR domains. Therefore, the reduced dominant-negative effect is likely a direct consequence of impaired co-assembly with endogenous MyD88.

However, direct experimental data specifically demonstrating this reduced co-assembly are not yet available, as discussed in Response-5 and Response-10. To better reflect the understanding that the reduced dominant-negative effect is likely a direct consequence of impaired co-assembly with endogenous MyD88, we have revised the statement on page 15, line 365 of the revised manuscript as follows:

*"Thus, the reduced dominant-negative effects are likely attributable to impaired intrastrand TIR-TIR interactions, which may **prevent proper co-assembly with endogenous MyD88.**"*

12. *Page 17 Line 435, "First, 434 TIRMyD88 rings were prepared on the AFM stage (Fig. 6a), and GST-TIRTLR2 was added" please add the concentration of the GST-TIRTLR2.*

Response-12: The concentration of GST-TIR$_{TLR2}$ used in the experiment described on page 18, line 460, has been specified in the revised manuscript as follows:

*"and GST-TIR$_{TLR2}$ was added **at a final concentration of 5 μM.**"*

13. *Page 18, Line 479: "In addition, the amount of GST-TIRTLR2 required for the promotion of self-assembly was only 5% that of TIRMyD88, strongly suggesting that GST-TIRTLR2 is required only at the initial stage of assembly." How did the author determine the amount of TIRMyD88 that works (5%)? Does lower amount also induce the self-assembly, and what is the limit?*

Response-13: Regarding the amount of GST-TIR$_{TLR2}$ used (5% relative to TIR$_{MyD88}$), we referred to the literature (Latty et al., *eLife*, 7, e31377 (2018); Deliz et al., *J. Cell Biol.*, 220, e202012071 (2021)), which indicates that approximately 20 MyD88 molecules can cluster beneath a single receptor complex. On this basis, a 1:20 receptor-to-adaptor stoichiometry corresponds to ~5% GST-TIR$_{TLR2}$, and this value was adopted as a roughly estimated yet physiologically motivated starting ratio.

Total protein concentrations and ratios were then adjusted to balance the assay sensitivity with a practical measurement window. Testing lower GST-TIR_{TLR2}:TIR_{MyD88} ratios revealed that values below 5% markedly delayed turbidity development; accordingly, we retained 5% as the minimal ratio that yielded a readily detectable and reproducible response. However, the apparent lower limit may depend on the assay format and detection window employed.

14. Page 46, line 1032: "For experimental samples of GST-TIRTLR2/TLR5, the expression vectors were transformed into Escherichia coli BL-21 (DE3) (Novagen)." Should be "The cells were transformed with the expression vectors."

Response-14: We thank the reviewer for this comment. The sentence has been revised to be grammatically correct.

Response to Reviewer #5

We would like to thank the reviewer for the careful assessment of our study and for their constructive comments, which have greatly improved our manuscript.

This manuscript presents a highly insightful investigation into the self-assembly of MyD88 and its interactions with Toll-like receptors (TLRs), using an impressive combination of cryo-electron microscopy (cryo-EM) and high-speed atomic force microscopy (HS-AFM). The study provides valuable structural and dynamic insights into the oligomerization of MyD88, which is crucial for understanding its role in signal initiation and immune response regulation.

The manuscript is well written, with a clear flow of ideas and a logical presentation of the experiments. The experimental design is robust and the results are convincing. The use of both cryo-EM and HS-AFM to observe the MyD88 oligomerization process is particularly commendable, as it offers complementary perspectives on the static and dynamic aspects of these molecular complexes. The findings significantly advance our understanding of MyD88's role in immune signaling and its potential implications in various pathologies including B-cell lymphomas and autoinflammatory diseases.

With a background in biophysics, my reviews focus more on the AFM experiments, the HS-AFM experiments are carefully executed, and the correlation between the HS-AFM and cryo-EM results strongly supports the authors' conclusions. The dynamic visualization of oligomerization using HS-AFM is particularly exciting, as it sheds light on the regulatory mechanisms governing this process.

One minor point for clarification: the authors mention (lines 199-200) that "in AFM, mechanical forces are applied to the specimen; thus, only molecular assemblies that are somewhat resistant to deformation are visualized." It would be beneficial if the authors could provide an estimation of the forces applied during imaging and discuss how these forces might influence the measured height values. Are the height values corrected for potential deformation, and how does this impact the interpretation of the structural data?

Response-1: We thank the reviewer for the positive assessment of our experiments and the constructive suggestions.

Two types of force were applied to the sample during the AFM observation. One is the force between the sample and the substrate, and the other is the force between the sample and the AFM tip.

The former force is difficult to estimate, but can sometimes be strong enough to break biomolecular complexes. For example, the subunits of cyclase-associated protein 1 (an actin-binding protein) and central spindlin (an unconventional kinesin) dissociate without tracing using an AFM tip under low-salt solution conditions (Kodera et al., *J. Cell Biol.*, 296, 100649 (2021); Davies et al., *PLoS Biol.*, 13, e1002121 (2015)). Similarly, in the present study, cylindrical fibers were not observed in the AFM images. Thus, we assumed that the interfilament interactions that promote cylinder formation were not sufficiently strong enough to withstand this force.

The latter force can be estimated to be approximately 20–30 pN based on the properties of the cantilever (i.e., its spring constant and quality factor of resonance) and the oscillation amplitude during observation (Garcia, R., *"Amplitude Modulation Atomic Force Microscopy"*, Wiley-VCH (2010)). Considering that Young's modulus of typical globular proteins is in the range of 0.5–5 GPa and that the contact area between the AFM tip and sample is on the order of a few nm^2, the deformation of a protein is estimated to be at most a few percent of the protein height. Because this small deformation was within the measurement error of the AFM, it could not be measured accurately. This is consistent with the fact that the height of the MyD88-TIR rings from HS-AFM imaging was almost identical to the results obtained with cryo-EM. Therefore, the AFM height values were not corrected, although they may include minor distortions.

In response to the reviewers' comments, the following sentence has been added to the main text on page 8, line 200 and the Methods section titled "High-speed AFM imaging":

"In AFM, two types of forces are applied to a sample; thus, only molecular assemblies that are resistant to these forces are observed. One such force is between the sample and the substrate, and the other is between the sample and the AFM tip. The former force is difficult to estimate, but it can sometimes be strong enough to break biomolecular complexes[28,29]. In contrast, the latter force can be estimated to be approximately 20–30 pN based on the properties and amplitude of the cantilever during observation[30], which is relatively small and unlikely to disrupt the assemblies. Therefore, it is most likely that the former force was responsible for preventing the formation of cylindrical fibers."

"The force applied between the AFM tip and the sample, which is estimated to be approximately 20-30 pN[30], may deform the protein structures to some extent. However, the deformation of a typical globular protein owing to this force is estimated to be, at most, a few percent of the protein height, which is smaller than the measurement error of HS-AFM. The AFM height values may contain such distortions; however, no corrections were made in the present study. Of note, the height of the TIRMyD88 rings from HS-AFM imaging was almost identical to that from the cryo-EM."

Another minor comment is related to Figure 4b: In the caption, the authors should display the individual data points and clearly explain what is represented (e.g., means ± standard deviation). This will help ensure transparency in the data presentation.

Response-2: In the figure, the luciferase activities of each mutant, which signify NF-κB activity, are presented as relative values compared to those of the internal control luciferase. Data are presented as mean values, with error bars representing the standard deviation from triplicate experiments. This information has been added in the revised manuscript.

The following sentence has been added to the main text on page 14, line 354.

"The data shown are the mean ± standard deviation from triplicate experiments, with individual data points displayed."

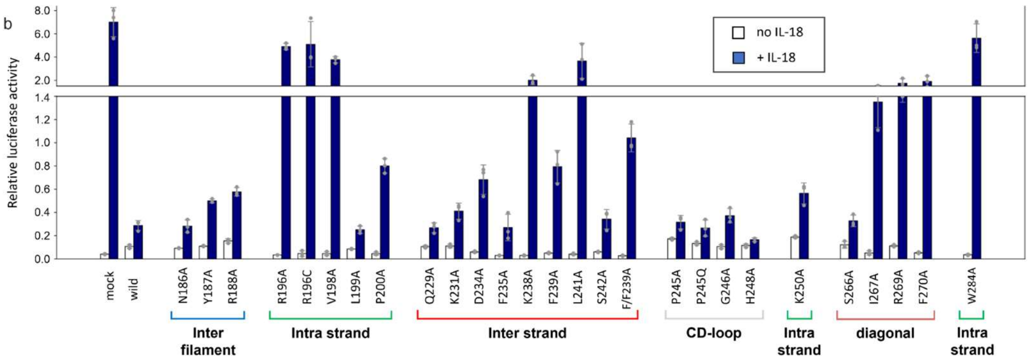
b
Relative luciferase activity
no IL-18
+ IL-18
mock
wild
N186A
Y187A
R188A
R196A
R196C
V198A
L199A
P200A
Q229A
K231A
D234A
F235A
K238A
F239A
L241A
S242A
F/F239A
P245A
P245Q
G246A
H248A
K250A
S266A
I267A
R269A
F270A
W284A
Inter filament
Intra strand
Inter strand
CD-loop
Intra strand
diagonal
Intra strand

February 27, 2026

Responses to Reviewers

Response to Reviewer #1

We sincerely thank Reviewer #1 for the careful evaluation of our manuscript and for the constructive comments.

The authors have revised their manuscript with additional experimental data and clearer text. The work provides important insights into the structural properties of the TIR domain. The authors propose that this structure facilitates the nucleation and assembly of death domain (DD) complexes. In particular, they clearly explain why they believe the MyD88-TIR structures explain how TIR receptors that do not require MAL trigger MyD88 oligomerization and Myddosome assembly.

We believe the major strength lies in a structural rationale linking TIR interfaces to DD assembly, particularly in the absence of MAL. The added data and improved clarity strengthen the central claims. The inclusion of GST–TIR–TLR5 experiments provides a valuable comparison to TLR2 (a receptor that signals with MAL).

Response-1: We thank the reviewer for the positive assessment and for the recognition of our structural rationale and the added GST–TIR–TLR5 experiments.

Major comments: The cellular validation of TIR-domain mutants remains the weakest part. To strengthen physiological relevance, we would like to see the authors test the mutants in the context of full-length MyD88 reconstituted into a MyD88-null line (e.g., immortalized MyD88−/− macrophages from BEI Resources), with appropriate expression controls. This system could then be used to compare signaling responses downstream of MAL-dependent TLRs (e.g., TLR2, TLR4) with those downstream of MAL-independent TLRs (e.g., TLR5), to contextualize the importance of the residues identified at the MyD88-TIR fiber interface.

Recommendation: Minor revisions. While additional cellular experiments would further strengthen the manuscript, they are not essential for publication and could be addressed in subsequent work by this or other groups.

Response-2: We thank the reviewer for this suggestion and agree that reconstitution of full-length MyD88 mutants into a MyD88-null background would provide strong physiological validation of the interfaces identified in our structural study. In the present work, however, our focus was the structural mechanism of receptor-triggered TIR–MyD88 assembly and its validation through dominant-negative assays; we believe these data support the relevance of the identified interfaces, while acknowledging that the suggested reconstitution system would offer an additional, valuable layer of validation. We consider this direction important and plan to pursue it in future work.
